# Chromosome-scale genome assembly of *Naematelia sinensis* (Jin Er mushroom) offers new insights into the distinct mating-type genomes feature and genome evolution

**DOI:** 10.3389/fmicb.2026.1669303

**Published:** 2026-04-02

**Authors:** Zhenhui Shen, Xiangying Luo, Linlei Yang, Yao Cao, Yukang Zeng, Qingqing Lu, Lingshan Zi, Rongchun Li

**Affiliations:** 1 Yunnan Junshijie Biotechnology Co. Ltd., Yunnan (Rare Edible Fungi) Enterprise Technology Center, Kunming (Edible Fungi) Enterprise Technology Center, Kunming, Yunnan, China; 2 Institute of Edible Fungi, Yunnan Agricultural University, Kunming, Yunnan, China

**Keywords:** basidiospore, comparative genomics, genome assembly, mating-type loci, *Naematelia sinensis*, whole-genome sequencing

## Abstract

*Naematelia sinensis* (Jin Er mushroom) is a gelatinous fungus of significant economic and medicinal value indigenous to China. As an obligate parasite that requires *Stereum hirsutum* for basidiomata formation, it serves as a distinctive model for investigating fungal-fungal interactions. Despite its potential as a source of bioactive polysaccharides, the scarcity of genomic data has impeded research into its secondary metabolism, cultivation, and underlying molecular mechanisms. We employed PacBio HiFi long-read sequencing and Hi-C chromatin conformation capture technology to generate the first chromosome-scale genome assemblies for four distinct mating types of *N. sinensis*. Genome assembly, annotation, and subsequent comparative genomic, phylogenomic, and gene family evolution analyses were conducted using standard bioinformatics pipelines. The assembled genomes span 20.73–20.82 Mb, resolved into 12 chromosomes with a contig N50 of 1.75–1.76 Mb, a GC content of 51.83–54.58%, and a BUSCO completeness of 96.8–97.1%. Annotation identified 19,390–19,612 repetitive sequences and 6,558–6,578 protein-coding genes. Comparative analysis with the related species *Tremella fuciformis* revealed extensive chromosomal rearrangements and inversions. The tetrapolar mating system was characterized, with mating-type loci A and B located on separate chromosomes, and elevated polymorphism was observed at the B locus. Phylogenomic analysis estimated the divergence of the *Naematelia* genus at approximately 154.88 million years ago and indicated a closer relationship between *N. sinensis* and *N.aurantialba* than with *N. encephala*. Gene family analysis highlighted frequent expansions and contractions throughout the evolutionary history of *Naematelia*, with an overall trend toward contraction. This study provides foundational genomic resources for *N. sinensis*. The high-quality genomes elucidate its genomes feature, diversity in mating systems, and evolutionary history. These resources will facilitate future research aimed at discovering bioactive compounds, developing cultivation strategies, and conducting broader studies on evolution in parasitic fungi.

## Introduction

Fungi play pivotal roles in ecosystems, biotechnology, and medicine, encompassing diverse research areas from evolution to biochemistry (Runnel et al., 2025; Case et al., 2025). Edible and medicinal mushrooms have garnered considerable attention due to their nutritional value and pharmacological properties. Many basidiomycetes produce complex fruiting bodies (termed basidiomata in technical contexts) and bioactive compounds that are not only significant to mycologists but also relevant to pharmacology, food science, and materials science (Garg, 2025). *Naematelia sinensis* (Tang and Yang, 2024), a distinctive golden gelatinous mushroom, exemplifies this significance through its extensive use in traditional Chinese medicine and cuisine. Investigating *N. sinensis* can elucidate fungal parasitism, medicinal compound biosynthesis, and mating genetics, offering interdisciplinary value for drug development, cultivation optimization, and fungal evolutionary research (Gandía and Garrigues, 2024).

Within the phylum Basidiomycota, the genus *Naematelia* Fr. (family *Naemateliaceae*) encompasses a group of fascinating jelly fungi, many of which are known for their intricate parasitic lifestyles. A prime example is *N. sinensis* (Tang and Yang, 2024), a rare edible and medicinal fungus endemic to China, colloquially known as ‘golden ear’ or ‘Jin Er’ (Chang and Miles, 2004). This species is distinguished by its vibrant golden, gelatinous basidiomes, which only develop through a specialized parasitic interaction with its host, the wood-decaying fungus *Stereum hirsutum* (Willd.) Pers. (Tang and Yang, 2024). The taxonomic placement of *N. sinensis* has been a subject of historical debate; it was once misidentified as *Tremella mesenterica* Retz ex Fr. (Luo et al., 1987) and later described as *Tremella aurantialba* (Bandoni and Zang, 1990). Subsequent multi-locus phylogenomic analyses placed it within the *Naemateliaceae*, renaming it *Naematelia aurantialba* (Bandoni and Zang, 1990) Millanes and Wedin (Liu et al., 2015). Most recently, a comprehensive study integrating morphology, phylogeny, and geography established the cultivated ‘Jin Er’ as a distinct species, *N. sinensis* (Tang and Yang, 2024) basidiomes.

*N. sinensis* is highly valued for its dual role as a gourmet food and a source of traditional medicine. Its purported health benefits, including antioxidant, anti-inflammatory, anti-tumor, and immunomodulatory activities, are largely attributed to a rich array of bioactive compounds such as polysaccharides, active proteins, terpenoids, phenolic acids, and flavonoids (Bandoni and Zang, 1990; Islam et al., 2016; Fan et al., 2017; Du et al., 2018; Liu et al., 2019). Polysaccharides, in particular, are a major component, constituting up to 74% of the dry weight of its basidiomes (Zhou et al., 2015). These properties have spurred its use in pharmaceuticals, functional foods, and high-end cosmetics (Yan et al., 2022). Despite significant progress in understanding its cultivation and medicinal properties (Wang et al., 2025), research has been critically hampered by the lack of a high-quality genomic reference. Previously available genomes for closely related taxa, such as *N. aurantialba*, are highly fragmented, consisting of hundreds of contigs and scaffolds (Sun et al., 2021; Shen et al., 2024). Such fragmented assemblies lack the completeness and contiguity required for advanced genetic studies, including comparative genomics, gene family evolution, and the identification of genes governing important traits (Wang et al., 2021).

The rapid evolution of sequencing technologies, particularly the combination of Pacific Biosciences (PacBio) single-molecule high-fidelity (HiFi) long reads and high-throughput chromosome conformation capture (Hi-C), now makes it possible to construct complete, chromosome-level genomes. This strategy has been successfully applied to assemble the genomes of numerous other economically important mushrooms, including *Agaricus bisporus* (J.E. Lange) Imbach (Sonnenberg et al., 2020), *Lentinula edodes* (Berk.) Pegler (Gao et al., 2022; Yu et al., 2022), *Sparassis latifolia* Y.C. Dai and F. Wu (Yang et al., 2021), *Wolfiporia hoelen* (Rumph.) Ryvarden and Gilb. (Li S. J. et al., 2022), *Stropharia rugosoannulata* Farl. ex Murrill (Li S. W. et al., 2022), *Dictyophora rubrovolvata* (M. Zang, D.G. Ji and S. Xu) D.G. Ji, S. Xu and R.H. Petersen (Ma et al., 2023), and *Tremella fuciformis* Berk. (Deng et al., 2023; Li et al., 2023). For *N. sinensis*, which exhibits a tetrapolar mating system common in Basidiomycota, this approach is particularly powerful. This system is governed by two unlinked mating-type (MAT) loci—one encoding homeodomain (HD) transcription factors and the other encoding pheromones and their receptors (P/R)—which control sexual compatibility between monokaryotic individuals (Sun et al., 2019; Cao et al., 2024; Shen et al., 2024). Assembling the genomes of multiple monokaryons representing different mating types provides a unique opportunity to investigate the genetic basis of this fundamental biological process.

The absence of a high-quality reference genome has been a significant barrier to understanding the fundamental biology and advancing the biotechnological application of *N. sinensis*. Therefore, the primary objective of this study was to generate the first chromosome-level genome assemblies for this important fungus. To achieve this, we addressed two central research questions: (1) What is the complete genomic architecture of *N. sinensis* at the chromosome level, and what structural and gene-level variations exist among its different mating-type monokaryons? (2) What is the evolutionary history of the *Naematelia* genus, and what genomic signatures underlie the unique parasitic lifestyle and metabolic capabilities of *N. sinensis*? In line with these questions, we formulated two key hypotheses. First, we hypothesized that the integration of PacBio HiFi long-read sequencing and Hi-C scaffolding would yield highly contiguous, chromosome-level assemblies for four distinct monokaryotic strains, enabling the detailed characterization of genomic structure, including the complex mating-type loci and variations in gene families such as Carbohydrate-Active enZymes (CAZymes) and Cytochrome P450s. Second, we hypothesized that comparative and evolutionary genomic analyses would robustly resolve the phylogenomic position of *N. sinensis* and reveal specific gene family expansions or contractions associated with its mycoparasitic nature and the biosynthesis of valuable secondary metabolites. This study provides a foundational genomic resource that will accelerate research into the genetics, evolution, and breeding of *N. sinensis* and its relatives.

## Materials and methods

### Fungal strains and culture conditions

The mature basidiomes of the Jin Er commercial cultivar were supplied by Yunnan Junshijie Biotechnology Co., Ltd., based in Yunnan, China. The dikaryotic strains of *Naematelia sinensis* (JSJ-J2F1001C) and *Stereum hirsutum* (JSJ-JEGJ-X18) were isolated from the basidiomes of the Jin Er cultivar and deposited in the China General Microbiological Culture Collection Center (CGMCC) under accession numbers CGMCC 41096 and CGMCC 19659, respectively. Monokaryotic basidiospores for genome sequencing were isolated from mature basidiomes using the spore-ejection method (Choi et al., 1999). Specifically, the basidiomes were surface-sterilized with 75% ethanol and suspended over a conical flask for 2 days to allow spore ejection. The ejected basidiospores were subsequently plated onto Potato Dextrose Agar (PDA) medium after gradient dilution. Single colonies were selected and preserved on agar slants at 4 °C. The number of nuclei and the microscopic morphology of yeast-like cells were examined using fluorescence microscopy (ECLIPSE Ts2R-FL, Nikon, Japan) and an optical microscope (Eclipse 80i, Nikon, Tokyo, Japan), respectively. Four monokaryotic strains (NS-27, NS-29, NS-45, and NS-58), characterized by distinct mating factors, were identified and screened as genomic sequencing materials following the methodology described by Shen et al. (2023). To obtain sufficient cell biomass for genomic DNA extraction, these strains were cultured on Potato Dextrose Agar (PDA: 200 g potato, 20 g dextrose, 18 g agar, 1,000 mL water) solid medium at 22 °C for 15 days. The induction medium (IDM) was primarily used to promote mycelial formation after the combination of two compatible monokaryotic basidiospore strains and to observe clamp connection formation. The preparation protocol for the induction medium is detailed in Reference (Cao et al., 2022). All reagents and chemicals were procured from Sigma-Aldrich (St. Louis, MO, USA).

### Genomic DNA extraction

After collection from the PDA solid medium, four monokaryotic basidiospores with distinct mating types were frozen and ground in liquid nitrogen. High-quality genomic DNA was extracted using a modified cetyltrimethylammonium bromide (CTAB) method optimized for fungal tissue (Doyle and Doyle, 1987). Approximately 0.2 g of frozen yeast-like cells was ground to a fine powder in liquid nitrogen using a mortar and pestle. The powdered tissue was transferred to a 50 mL Falcon tube and mixed with 20 mL of CTAB extraction buffer (2% CTAB, 100 mM Tris–HCl pH 8.0, 20 mM EDTA, 1.4 M NaCl, 1% PVP-40, and 0.2% *β*-mercaptoethanol). The mixture was incubated at 65 °C for 60 min with occasional gentle inversion. After incubation, the sample was extracted with an equal volume of chloroform:isoamyl alcohol (24:1) by inverting the tube gently for 10 min. The mixture was centrifuged at 12,000 rpm for 15 min at 4 °C to separate the phases. The upper aqueous phase was transferred to a new 50 mL tube, and an equal volume of isopropanol was added to precipitate the DNA. The DNA was spooled out with a glass rod and washed with 70% ethanol. The DNA pellet was air-dried and resuspended in 500 μL of TE buffer (10 mM Tris–HCl, 1 mM EDTA, pH 8.0). RNA was removed from the samples using RNase A (Leagene, Beijing, China) at a concentration of 10 μg/mL. The quality and quantity of the extracted DNA were assessed using a NanoDrop 2000 spectrophotometer (NanoDrop Technologies, Wilmington, DE, USA), the Qubit dsDNA HS Assay Kit on a Qubit 3.0 Fluorometer (Life Technologies, Carlsbad, CA, USA), and electrophoresis on a 0.8% agarose gel.

### Genome sequencing and assembly

The extracted genomic DNA was used for construction of both PacBio genome sequencing library and Illumina short read sequencing library. Short-read libraries with an insertion size ranging from 300 bp to 400 bp were prepared by the Beijing Genomics Institute (BGI) using the Optimal DNA Library Prep Kit (Shenzhen, China). The quality and size of the library’s insertion fragments were assessed using the Agilent Bioanalyzer 2,100 (Agilent Technologies, Santa Clara, CA, USA). Subsequently, the library was sequenced on the DNBSEQ-G400 platform to generate paired-end sequence data. For each strain, a high-fidelity (HiFi) long-read library was prepared using the Pacific Biosciences (PacBio) SMRTbell Express Template Prep Kit 2.0. Genomic DNA was sheared to an average size of ~30 kb using a g-TUBE device (Covaris). Size-selected DNA fragments were end-repaired, A-tailed, and ligated with PacBio SMRTbell adapters. The ligated products were size-selected again to enrich for fragments of the desired size (approximately 20–30 kb). Each library was quantified using qPCR with the SMRTbell Template Quantification Kit 2.0 and diluted to a concentration of 10 nM. The libraries were then loaded onto a PacBio Sequel IIe instrument and sequenced with P6-C4 chemistry to generate long reads. Approximately 100 × coverage of high-quality sequencing data was obtained for each strain, yielding an average read length of ~20 kb.

The raw BGI short reads were preprocessed using SOAPnuke (version 2.1.0) (Chen et al., 2018), with the following parameters: “-n 0.001 -l 20 -q 0.4 --adaMis 3 --rmdup --minReadLen 150”. Subsequently, the genome size and heterozygosity were estimated using Genomescope 2 with a 21 k-mer (Ranallo-Benavidez et al., 2020). The raw sequencing data generated by the PacBio Revio platform underwent quality control via SMRT Link (version 11.1.0) (Chin et al., 2013), during which low-quality reads (less than 500 bp) and adapter sequences were removed to produce high-quality subreads. After obtaining HiFi reads, two independent *de novo* assemblies were generated for comparative analysis: one using Hifiasm v. 0.14-r312 (Cheng et al., 2021) with default parameters, and the other using NextDenovo v2.5.0^1^ with default parameters. Finally, Pilon (version 1.18) (Walker et al., 2014) was employed to refine the assembly based on the short reads.

Based on the evolutionary information of single-copy orthologous genes across all fungal species, the Benchmarking Universal Single-Copy Orthologs (BUSCOs, http://busco.ezlab.org, version 5.2.2, basidiomycota_odb10) (Simão et al., 2015) was employed to evaluate the integrity of the genome assembly. Additionally, Merqury (version 1.3) (Rhie et al., 2020) was utilized to assess the k-mer completeness and the consensus quality value (QV) of the genome assembly.

### Hi-C sequencing and analysis

Hi-C libraries were constructed following a standard *in situ* protocol. Intact nuclei were cross-linked with 1% formaldehyde to preserve three-dimensional chromatin contacts. Following DpnII digestion, fragment ends were repaired, biotinylated, and proximity-ligated using T4 DNA ligase to generate chimeric circles. Reverse cross-linking, shearing to 300–500 bp, and streptavidin-mediated enrichment of biotin-tagged junctions were performed prior to adapter ligation and limited-cycle PCR. The resulting libraries were quantified, size-verified, and subjected to paired-end 150-bp sequencing on the DNBSEQ-G400 platform (MGI, Wuhan, China) to achieve approximately 100 × physical coverage. Raw reads were filtered using SOAPnuke (version 2.1.0). Hi-C clean reads were aligned to the reference genome using Juicer (version 1.6) (Durand et al., 2016a), and preliminary clustering and orientation of the data were performed using 3D-DNA (version 180,922) (Dudchenko et al., 2017). Valid data obtained after alignment by Juicer were further processed using 3D-DNA JuiceBox (version 1.11.08) (Durand et al., 2016b) for automated clustering, sorting, and orientation, with visual error correction.

### Genomic component prediction and functional annotation

RepeatMasker (version 4.1.1) (Flynn et al., 2020) was employed for the prediction of repetitive sequences, while tRNAscan-SE (version 1.3.1) (Chan et al., 2021) and Rfam (Kalvari et al., 2021) were utilized for the identification of tRNA and rRNA sequences. To predict coding genes, a combination of Augustus (version 3.4.0) (Stanke and Morgenstern, 2005), glimmerHMM (version 3.0.1) (Majoros et al., 2004), GeneMark-ES (version 4.3.5) (Majoros et al., 2004), exonerate v2.2.0,^2^ PASA, and EvidenceModeler (Haas et al., 2008) was applied using three distinct strategies: (a) *de novo* prediction, (b) homology-based search, and (c) transcriptome-assisted annotation. Functional annotations of the predicted genes were performed against multiple databases, including Gene Ontology (GO) (Consortium, 2019), Kyoto Encyclopedia of Genes and Genomes (KEGG) (Kanehisa and Goto, 2000), NCBI Non-redundant protein database (NCBI nr), SwissProt, and Carbohydrate-Active enzymes (CAZymes) (Cantarel et al., 2009), utilizing BLAST+ (Camacho et al., 2009) and DIAMOND (Buchfink et al., 2015). The E-value threshold was set to <1 × 10^−5^, and the minimal alignment length percentage was 40%. Additionally, Diamond 2.9.0 (e-value > e−5) in conjunction with the Hmmer package was used for the prediction of P450s and the annotation of target protein sequences. Reference P450 sequences for cluster analysis were retrieved from the Fungal Cytochrome P450 Database.^3^

### Identification of mat-genes

Based on gene function annotation information retrieved from databases such as Gene Ontology (GO), KOG, and Swiss-Prot, the *HD1* and *HD2* gene sequences were identified, and the position of the A mating-type locus was determined. According to the gene function annotation results, the pheromone receptor gene (*STE3*) and the pheromone precursor gene (*phB*) associated with the B (P/R) mating-type locus were further investigated. Given that pheromone precursors are typically located within approximately 10 kb of the pheromone receptor genes, the location of the pheromone receptor gene was established. Subsequently, to identify pheromone precursors, the 10-kb flanking regions upstream and downstream of each *STE3* receptor gene were extracted and analyzed for open reading frames (ORFs) using the NCBI Open Reading Frame Finder. The predicted ORFs were then translated in silico and filtered for peptides that (i) terminate with a CaaX prenylation motif, where C represents cysteine, the two central residues (a) are aliphatic (A, V, L, I, G), and the C-terminal residue (X) is A, S, M, Q, or C, and (ii) contain the conserved N-terminal signatures AF and ER (Clarke, 1992; Bao, 2019; Shen et al., 2024). Collinearity analysis of the upstream and downstream genes at the mating-type loci A and B in *N. sinensis* was conducted using ChromoMapper software.^4^

### Comparative genomics analysis

The all-versus-all BLASTP method (E-value < 1 × 10^−5^) was employed to identify orthologous genes across four *N. sinensis* genomes. Single nucleotide polymorphisms (SNPs) and insertions/deletions (InDels) were detected based on genomic alignment results of shared genes between the following strain pairs: NS-27 and NS-45, as well as NS-29 and NS-58. These analyses were conducted using the MUMmer (Kurtz et al., 2004) and LASTZ (Altschul et al., 1990; Chiaromonte et al., 2002) tools. To detect genomic structural variation, we conducted a genome-wide collinearity analysis. Syntenic paralogous blocks were identified using MCSCAN (Wang et al., 2012) by comparing our genomes with the publicly available genomes of *N. sinensis* strain NX-20 and *T. fuciformis* strains TWW01-AX and Tr01. All these reference genomes correspond to monokaryotic isolates with complete gene annotations deposited in NCBI. To identify strain-specific genes, we first extracted coding sequences that lacked aligned counterparts between pairs of strains. Each putative unique gene set was then validated at the protein level to exclude assembly or annotation artefacts. Briefly, strain-specific peptides were retrieved with seqkit v2.3.0, reciprocal BLASTP (e-value ≤ 1e-5) was performed against the proteome of every partner strain, and hits ≥ 1 were discarded. Only proteins with zero reciprocal matches were retained as bona-fide strain-specific genes. KEGG and GO enrichment analyses were performed utilizing the OmicShare tools.^5^ Significantly enriched pathways and GO terms among rearranged genes were compared against syntenic genomes via a hypergeometric test. The resulting *p*-values were adjusted using false discovery rate (FDR) correction, with an FDR threshold of ≤ 0.05. Pathways and GO terms satisfying this criterion were designated as significantly enriched.

### Phylogenomic analysis

To explore the evolutionary dynamics of *N.sinensis*, the genome sequences of additional 19 fungal species were downloaded from NCBI for phylogenomics analysis (Supplementary Table S13). The single-copy orthologous genes of these 23 species were identified using OrthoFinder (Steve Kelly Lab, Oxford, UK) (Emms and Kelly, 2015). Subsequently, the amino acid sequences of these single-copy genes were aligned with MAFFT (version 7.505) using default parameters (Madeira et al., 2022), followed by the screening of conserved regions with Gblocks (Castresana, 2000) and concatenation into supergenes via Phylosuite (Zhang et al., 2020). Maximum likelihood phylogenomic trees were constructed using RAxML (version 8.2.12) with LG + I + G4 + F model, statistical support values were obtained using nonparametric bootstrap with 1,000 replicates (Stamatakis, 2014). While divergence times were estimated using the MCMC tree module in the PAML software package with the following parameters: clock = 3 (correlated rates); ndata = 1; seqtype = 2; model = 0 (JC69); aaRatefile = wag.dat; burnin = 1,000,000; sampfreq = 10; nsample = 500,000 (Sanderson, 2003). For molecular clock calibration, the divergence time between the two outgroup fungi, *Ustilago hordei*(*Ustilaginomycotina*) and *Wallemia mellicola*(*Wallemiomycotina*), was used as a calibration constraint, with an estimated range of 406–501 million years ago (Mya) obtained from the TimeTree database. The expansion and contraction of gene families across the 23 fungal species were predicted using the Computational Analysis of gene Family Evolution (CAFE version 4.2.1) software (De Bie et al., 2006) with the following parameters: a cut-off *p*-value of 0.05; number of random samples = 1,000; the lambda value to calculate birth and death rates.

### Count of chromosome number in *N. sinensis*

Monokaryotic basidiospores were harvested during the logarithmic growth phase and subsequently treated with 5 g/L colchicine for 2.5 h. The cells were then incubated at 4 °C for 3 h, followed by fixation with formalin at room temperature overnight. After fixation, the cells were stained with 20 mg/L DAPI and analyzed using confocal microscopy (Carl Zeiss AG, Oberkochen, Germany).

## Results

### Screening and analysis of *N. sinensis* sequencing strains

**Figure 1:**
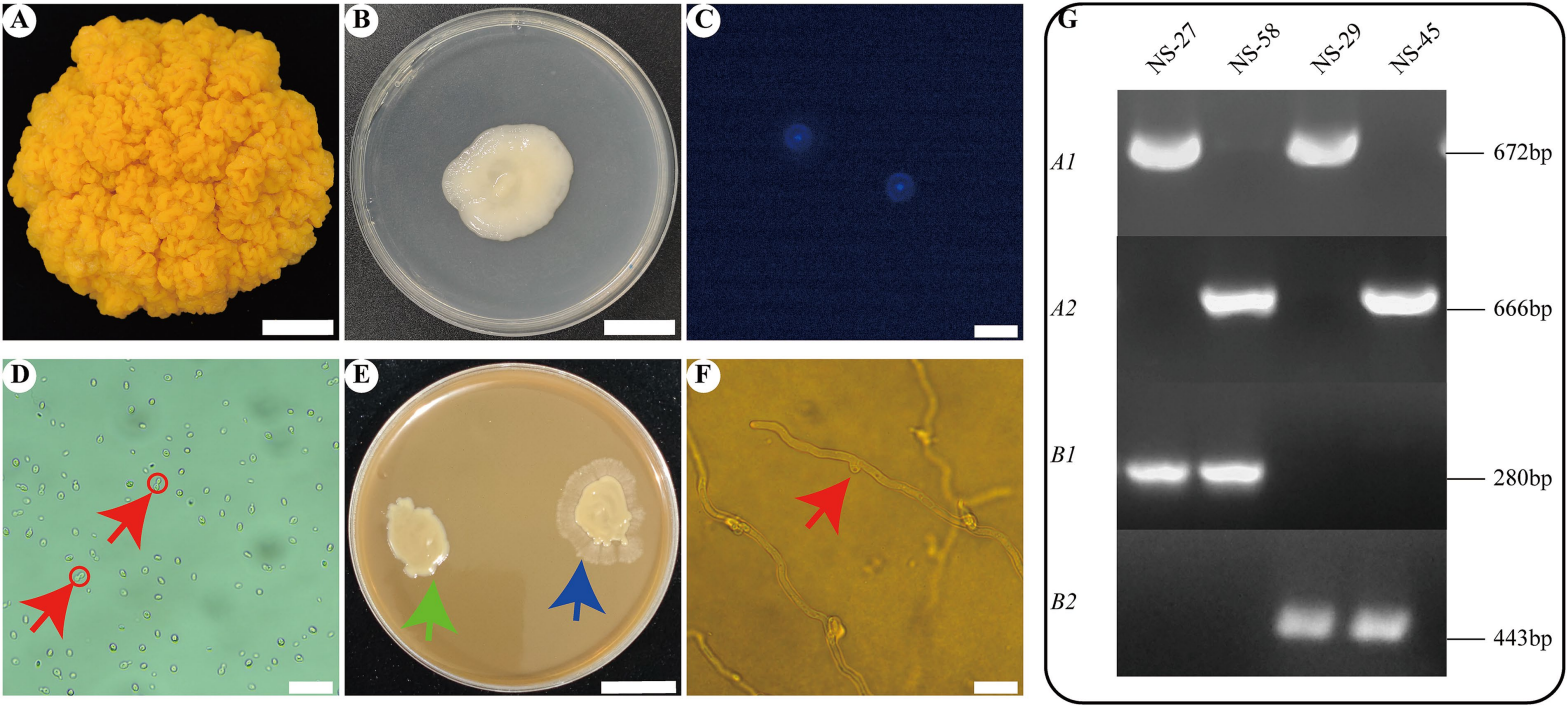
The phenotype, microscopic morphology, and mating type of four *N. sinensis* strains. **(A–F)** Represent the fruiting body of *N. sinensis*, the cultivation of basidiospores on PDA, the observation of nuclei in basidiospores, the microscopic morphology of yeast-like cell reproduction in basidiospores, the morphology of hybrid strains under induction medium, and the clamp connections of mycelium in hybrid strains, respectively. Scale bars: 2 cm (**A**,**B**,**E**), 20 μm (**C**,**D**,**F**). **(G)** Mating type identification of 4 strains.

Mature fruit bodies of *N. sinensis* were successfully obtained from the *N. sinensis* strain JSJ-J2F1001C, kindly provided by Yunnan Junshijie Biotechnology Co. Ltd. (Figure 1A). Basidiospores were harvested using a standardized basidiospore collection method, and yeast-like conidia were subsequently cultured on PDA solid medium (Figure 1B). These conidia underwent asexual reproduction via budding (as indicated by the red arrows in Figure 1D). Fluorescence microscopy confirmed that the basidiospores of *N. sinensis* were monokaryotic (Figure 1C). Based on preliminary compatibility analyses among different monokaryotic strains of *N. sinensis* (Cao et al., 2024), four strains with distinct mating factors—NS-27, NS-29, NS-45, and NS-58—were selected for subsequent genome sequencing. To validate the compatibility and mating types of these strains, pairwise mating experiments were performed, demonstrating that the combinations of NS-27 with NS-45 and NS-29 with NS-58 resulted in sexually compatible hybrids (Supplementary Table S1). After cultivating these two compatible hybrid strains in an induction medium for 15 days, faint mycelial growth was observed (Figure 1E, indicated by blue arrows), whereas incompatible strains exhibited no mycelial growth (Figure 1E, indicated by green arrows). Optical microscopy revealed clamp connections within the mycelium (Figure 1F, indicated by red arrows). Mating type primers (Shen et al., 2023) were employed to determine the mating types of the four strains, confirming that the mating types of NS-27, NS-29, NS-45, and NS-58 were A1B1, A1B2, A2B2, and A2B1, respectively (Figure 1G). Furthermore, DNBSEQ-G400 sequencing was utilized for genomic analysis. As shown in Supplementary Figures S1A–D, the genome sizes of the four *N. sinensis* strains ranged from approximately 21 to 22 Mb, with a heterozygosity ratio of 0.23 to 0.27% at a k-mer value of 21. Collectively, these findings confirmed the successful isolation of four monokaryotic strains with distinct mating factors, which were suitable for further monokaryotic genome sequencing and assembly.

### Genome sequencing and assembly

In this study, we conducted the genome assembly of four monokaryotic strains of *N. sinensis* using Hifiasm and Nextdenovo. The results demonstrated that Hifiasm generated assemblies with a higher number of contigs (ranging from 55 to 103) and variable genome sizes (from 18.76 to 20.40 Mb), with an N50 ranging from 1,762,575 to 2,341,609 bp. In contrast, Nextdenovo produced fewer contigs (13 per strain), with more consistent genome sizes (20.73 to 20.82 Mb) and an N50 ranging from 1,749,571 to 1,765,968 bp (Supplementary Table S2). Overall, Nextdenovo exhibited superior performance compared to Hifiasm in assembling the *N. sinensis* genome.

In the four *N. sinensis* genomes, 13 contigs were assembled in each, which is two fewer than the 15 reported for the reference strain NX-20. The genome sizes and lengths of the largest contigs were comparable among all five strains, averaging approximately 21 Mb and 2.54 Mb, respectively. The average N50 of the new assemblies was 1,758,927 bp, which is approximately 55 kb shorter than that of NX-20. The GC content averaged 53.65%. The annotation of NX-20 combined *de novo* (Augustus) and homology-based evidence from N. encephala but did not incorporate RNA-seq data. In contrast, the four genomes reported here were annotated using an integrative pipeline that incorporated comprehensive transcriptome evidence (see Materials and methods). Consequently, the new assemblies contain an average of 6,564 predicted protein-coding genes, which is 704 more than the 5,860 genes reported for NX-20, and the total coding sequence accounts for 48.24% of the genome, approximately 6 percentage points higher than NX-20. These increases likely reflect the enhanced sensitivity provided by transcriptome support rather than true biological expansion (Table 1).

**Table 1:** Statistics of *N. sinensis* genomes assembly and gene prediction.

Feature	NS-27	NS-29	NS-45	NS-58	Average value	NX-20
Genome size (Mb)	20.73	20.80	20.77	20.82	20.78	20.99
Number of contigs	13	13	13	13	13	15
Max length (bp)	2,543,173	2,539,498	2,537,851	2,554,102	2,543,656	2,546,384
Contig N50 (bp)	1,757,723	1,765,968	1,762,447	1,749,571	1,758,927	1,814,705
GC contents (%)	54.58	54.49	51.83	53.69	53.65	56.42
Number of genes	6,578	6,558	6,560	6,562	6,564	5,860
Average gene length (bp)	2467.37	2459.77	2451.45	2463.23	2460.45	-
Average cds length (bp)	1602.73	1602.44	1600.82	1599.81	1601.45	1,534
Average number of exons per gene	6.83	6.82	6.82	6.63	6.775	-
Average exon length (bp)	234.71	234.91	234.64	234.31	234.64	-
Average intron length (bp)	98.52	98.37	96.41	99.02	98.08	-
Gene total length (Mb)	10,542,758	10,505,916	10,502,560	10,499,200	10,512,609	8,989,977
Gene length/Genome (%)	48.50	48.17	48.22	48.09	48.24	42.81
Reference	This study	This study	This study	This study		Sun et al. (2021)

Subsequently, BUSCO analysis and Merqury evaluation were performed to assess the quality of the genome assemblies of the four *N. sinensis* strains. A total of 1,764 BUSCOs v5.2.2 (basidiomycota_odb10) were identified across the four genome assemblies, with the complete BUSCO rates being 97.10, 97.00, 96.90, and 96.80% for NS-27, NS-29, NS-45, and NS-58, respectively (Supplementary Table S3). When the same assemblies were re-evaluated with BUSCOs v6.0.0 against the updated basidiomycota_odb12 lineage set (2,409 BUSCO groups), completeness remained consistently high (96.1–96.3%), while the proportion of duplicated BUSCOs was negligible (< 0.1%) and missing genes accounted for only 2.9–3.1%, confirming the robustness and completeness of the four genome assemblies across different BUSCO versions and lineage databases (Supplementary Table S3). The Merqury evaluation revealed k-mer completeness ranging from 99.62 to 99.75%, with consensus quality values (QV) ranging from 54.72 to 57.51, confirming a substantial improvement in both the accuracy and completeness of the four genome assemblies (Supplementary Table S4). Comparison with the genome assemblies of *Tremellaceae* species available in the NCBI database demonstrated that our assembly results surpass those of these species (Table 2).

**Table 2:** Assembly summary statistics compared to other mushrooms of *Tremellales*.

Species	NCBI BioProject	Total length (Mb)	GC%	Contigs	N50 length (bp)	BUSCOs	Fragmented	Missing
*N. sinensis_*NS-27	PRJNA1264744	20.73	54.58	13	1,757,723	97.10%	1.0%	1.9%
*N. sinensis_*NS-29	PRJNA1264744	20.80	54.49	13	1,765,968	97.00%	1.0%	2.0%
*N. sinensis_*NS-45	PRJNA1264744	20.77	51.83	13	1,762,447	96.90%	1.1%	2.0%
*N. sinensis_*NS-58	PRJNA1264744	20.99	53.69	13	1,814,705	96.80%	1.1%	2.1%
*N.aurantialba. NX*-20	PRJNA772294	20.99	46.8	15	1,825,336	92.0%	1.4%	6.6%
*N. encephala* 68–887.2	PRJNA330699	19.79	49.3	151	209,500	85.5%	3.4%	11.1%
*T. mesenterica* DSM 1558	PRJNA225529	28.64	46.8	484	123,767	92.0	1.4%	6.6%
*T. fuciformis* Tr26	PRJNA281519	23.64	57.0	3,502	18,448	92.4%	1.4%	6.2%
*T. fuciformi* TWW01-AX	PRJNA924222	28.4	56.5	19	228,235	93.1%	0.6%	6.3%

**Figure 2:**
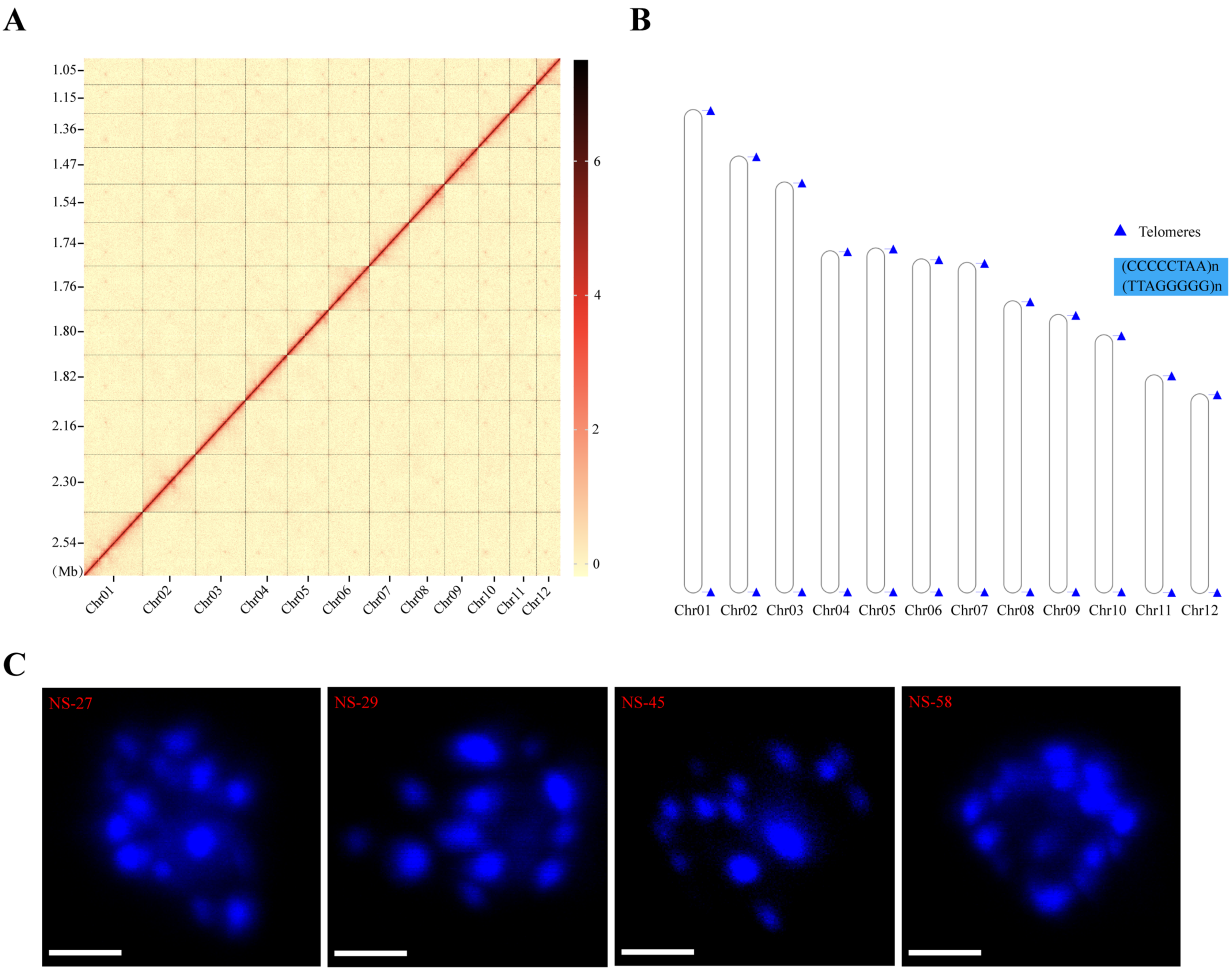
Assembly of the chromosomal-scale genome. **(A)** Hi-C interaction heatmap of all bins in the NS-27 strain. The numbers on the left represent the chromosome length, the color in the figure from lighter to darker indicates the increase in the intensity of the interaction, and the darker the stronger the interaction. **(B)** Telomeres were labeled on the chromosomes. The blue triangles represent the telomere. **(C)** Chromosome number of *N. sinensis*. Scale bar = 2 μm.

High-throughput chromosome conformation capture (Hi-C), a massively parallel DNA sequencing technique, facilitates the generation of chromosome-length scaffolds and highly contiguous genome assemblies. In this study, we employed Hi-C technology to enhance genome assembly by leveraging paired-end sequencing data obtained from the DNBSEQ platform. Following quality filtering and removal of duplicate reads, approximately 2.44–2.57 Gb (118–125 × coverage) of valid reads were utilized to refine the contig sequences derived from the aforementioned HiFi data. Through a series of computational steps including fragmentation, clustering, sorting, orientation, and correction using 3D-DNA and JuiceBOX tools, 13 HiFi contigs were successfully clustered into 12 chromosome groups. Additionally, a circular mitochondrial genome (Hic_scaffold_7) was assembled. The chromosomes exhibited varying lengths, with the largest spanning approximately 2.54 Mb and the smallest approximately 1.05 Mb (Supplementary Table S5). A heatmap generated using Hi-C data confirmed that all bins could be assigned to the 12 chromosomes (Figure 2A and Supplementary Figures S2A–D). Within each group, the interaction intensity at diagonal positions exceeded that at off-diagonal positions, indicating strong interactions between adjacent sequences in the Hi-C assembly results. Conversely, the interaction signals between non-adjacent sequences (off-diagonal positions) were weak, consistent with the principles of Hi-C-assisted genome assembly. Notably, no significant noise (e.g., stronger interaction intensities outside the diagonal) was observed, confirming the satisfactory quality of the genome assembly. Furthermore, telomeres were detected at both ends of all 12 chromosomes, characterized by the 5′ telomere repeat monomer (CCCCCTAA)n and the 3′ telomere repeat monomer (TTAGGGGG)n (Figure 2B and Supplementary Table S6). To validate the chromosome count, we conducted microscopic observations of four *N. sinensis* individuals under confocal microscopy (Figure 2C). Collectively, these findings demonstrated the successful construction of four chromosomal-level *N. sinensis* genomes, each comprising 12 chromosomes.

### Genomic component prediction and functional annotation

Repetitive sequences account for approximately 4.19, 4.27, 4.46, and 4.63% of the NS-27, NS-29, NS-45, and NS-58 genomes, respectively (Supplementary Table S7). The majority of these repeats were classified as simple repeats and long-terminal repeats (LTRs), comprising 2.50–2.52% and 1.02–1.56% of the four genomes, respectively (Supplementary Table S7). Among the LTRs, the most abundant subtype is Gypsy, which constitutes 0.88–1.29% of the genomes. Noncoding RNAs (ncRNAs), a class of RNA molecules that perform diverse biological functions without encoding proteins, directly influence life activities at the RNA level. The results of ncRNA analysis in the four *N. sinensis* genomes are summarized in Supplementary Table S8. With respect to RNA content, the four genomes collectively predicted 12–18 rRNAs, 4 snRNAs, and 25 tRNAs. Additionally, a total of 6,578, 6,558, 6,560, and 6,562 protein-coding gene models were predicted for the respective genomes (Table 1). To elucidate the functional roles of these protein-coding genes, we conducted functional annotations against the NCBI NR, Pfam, KEGG, SwissProt, GO, COG, TCDB, and CARD databases. In total, 6,477 (98.46%), 6,503 (99.16%), 6,497 (99.04%), and 6,494 (98.96%) genes were successfully annotated across eight databases for the four genomes, respectively (Supplementary Table S9).

### Collinearity analysis of *N. sinensis* strains and *T. fuciformis* strains

**Figure 3:**
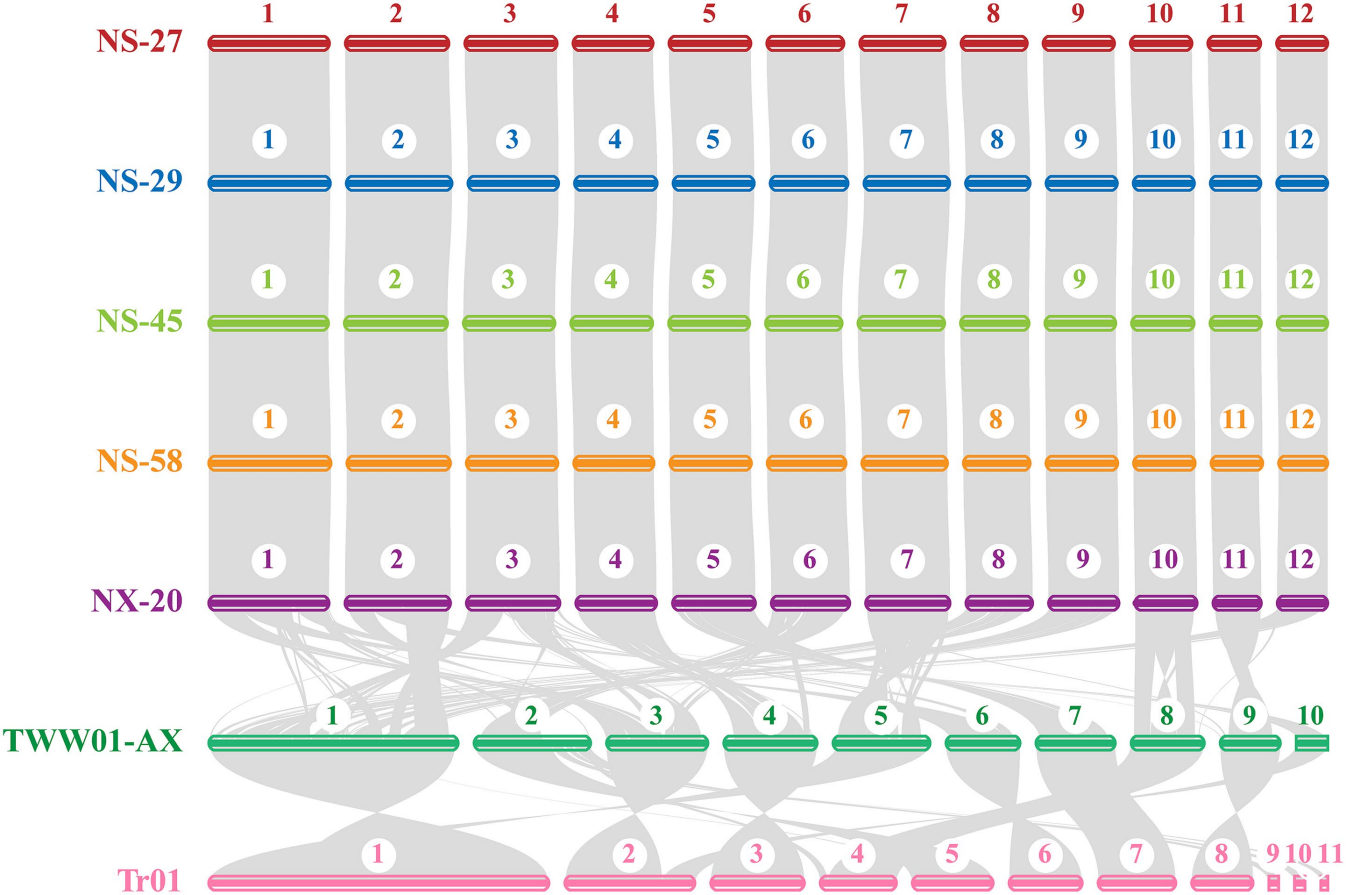
The genome colinearity analysis of the seven strains (including five *N. sinensis* strains and two *T. fuciformis* strains) using the protein-coding genes.

We retrieved the publicly accessible genome sequences and annotation data for a monokaryotic strain of *N. sinensis* and two monokaryotic strains of *T. fuciformis* from NCBI (Supplementary Table S10). Synteny genes among seven strains were identified through collinearity analysis performed using MCSCAN, and a collinearity map was generated to visualize the relationships among these strains (Figure 3A). Based on the collinearity analysis results, these four *N. sinensis* strains exhibited strong synteny with the *N. sinensis* NX-20 strain. Additionally, a high level of collinearity was observed between the two *T. fuciformis* strains. Collinearity comparisons between *T. fuciformis* and *N. sinensis* revealed several inversions and rearrangements in homologous regions. For instance, a large inversion was detected in Chr10 of *N. sinensis* and Chr8 of TWW01-AX, as well as in Chr7 of *N. sinensis* and Chr5 of TWW01-AX. Rearrangements of varying degrees were also observed on other chromosomes, such as chromosome 1 of the TWW01-AX strain showing collinearity with chromosomes 1, 2, 3, 6, 8, 9, and 12 of *N. sinensis*. Furthermore, a unique region was identified on chromosome 2 of the *T. fuciformis* TWW01-AX strain. Genes located in non-syntenic regions might be associated with morphological development and environmental adaptation.

### Comparative analysis of four *N. sinensis* strains

The pairwise synteny analyses of the genomes of four *N. sinensis* strains were performed based on nucleic acid sequences. The results demonstrated that the syntenic regions between two sexually compatible strains, NS-27 and NS-45, were highly conserved, with only minor structural variations observed in a few regions. For instance, non-syntenic regions were identified in the middle of Chr01 and Chr03, along with a small inversion at the terminus of Chr10 (Supplementary Figure S3A). The synteny analysis between NS-29 and NS-58 revealed that all 12 chromosomes exhibited highly conserved syntenic regions (Supplementary Figure S3B). Comparative synteny analyses among other strains further indicated that the genomes of the four *N. sinensis* strains, which possessed distinct mating types, were highly conserved, with no significant structural variations detected (Supplementary Figure S4). This suggests that the *N. sinensis*strains analyzed in this study may have relatively conserved genomic features.

**Figure 4:**
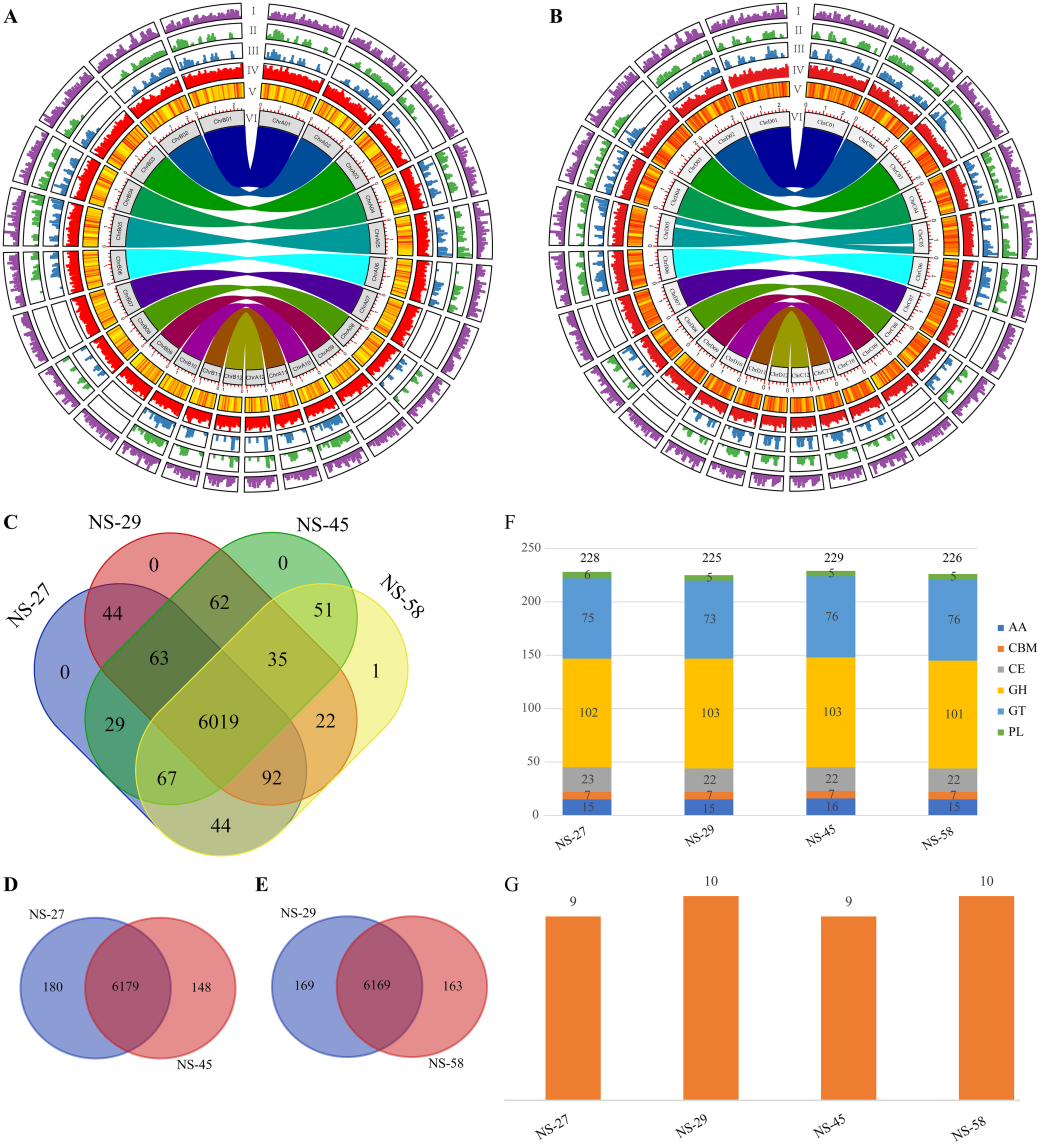
Genomic features of sexual compatible strains, statistical data of unique genes, CAZymes, and cytochrome P450 in the four *N. sinensis* genomes. **(A)** Genome circus plot of sexual compatible strains NS-27 (left side) vs. NS-45 (right side); **(B)** Genome circus plot of sexual compatible strains NS-29 (left side) vs. NS-58 (right side). The tracks from outside to inside: transposable elements density, SNP density, InDel density, gene density, GC density and haplotype chromosome were calculated in 50 kb windows. **(C)** VN diagram of shared and unique genes among four *N. sinensis* genomes; **(D)** VN diagram of shared and unique genes between NS-27 and NS-45 genomes; **(E)** VN diagram of shared and unique genes between NS-29 and NS-58 genomes; **(F)** The number of CAZymes genes in the four *N. sinensis* strains (AA, auxiliary activity; CBM, carbohydrate-binding module; CE, carbohydrate esterase; GH, glycoside hydrolase; GT, glycosyl transferase; PL, polysaccharide lyase); **(G)** The number of cytochromers P450 genes in the four *N. sinensis* strains.

Upon assembly and circular plot analysis of the genomes of four strains, it was observed that repetitive sequences and genes were relatively evenly distributed across the 12 chromosomes. In a comparative analysis of two sets of sexual compatible strains (NS-27 vs. NS-45 and NS-29 vs. NS-58), 67,419 (7,171) and 74,898 (8,302) SNPs (InDels) sites were identified, respectively. Notably, with the exception of the lower distribution observed in Chr07, Chr09, and the downstream region of Chr03, these variant sites demonstrated varying degrees of enrichment across other chromosomes (Figures 4A,B).

Using OrthoFinder software and Venn diagram analysis, a comprehensive investigation was conducted into the shared and unique genes among four distinct mating-type strains of *N. sinensis*. Following rigorous validation through reciprocal BLASTP searches to exclude assembly and annotation artifacts, the findings revealed that these four strains collectively share 6,019 orthologous genes. Notably, with the exception of strain NS-58, which harbored one unique gene, no unique genes were identified in the other strains when analyzed collectively (Figure 4C). However, a comparative analysis of sexually compatible strains revealed a striking pattern that could potentially reflect functional specialization rather than direct complementation. The NS-27 and NS-45 strains shared 6,179 common genes, while possessing 180 and 148 validated unique genes, respectively (Figure 4D). Similarly, the NS-29 and NS-58 strains shared 6,169 common genes, with 169 and 163 validated unique genes, respectively. To explore potential functional implications of these unique genes, we performed detailed functional analysis of representative examples. For instance, NS-27 possesses unique genes encoding a protein phosphatase 2A regulatory subunit (involved in cell cycle regulation) and a formin-like protein (essential for actin nucleation), while its compatible partner NS-45 harbors unique genes for a SNARE protein (critical for vesicle fusion) and a glycosyl hydrolase (involved in carbohydrate metabolism) (Supplementary Table S11). This reciprocal distribution of unique genes between compatible pairs raises the possibility that these genetic differences may contribute to functional specialization in the dikaryotic state. This hypothesis, generated from genomic data, awaits future validation through transcriptomic, proteomic, and functional assays.

A comparative analysis of the CAZyme families among the four strains of *N. sinensis* identified a total of 225 to 229 CAZymes distributed across these strains. No significant variation was observed in the gene counts within the CAZyme families among the four strains, with the majority of genes being classified into glycoside hydrolases (GHs), glycosyl transferases (GTs), and carbohydrate esterases (CEs) (Figure 4F). Additionally, an analysis of the P450 gene family in the four strains revealed that only 9 to 10 gene members were detected (Figure 4G).

### Analysis of mating-type genes

**Figure 5:**
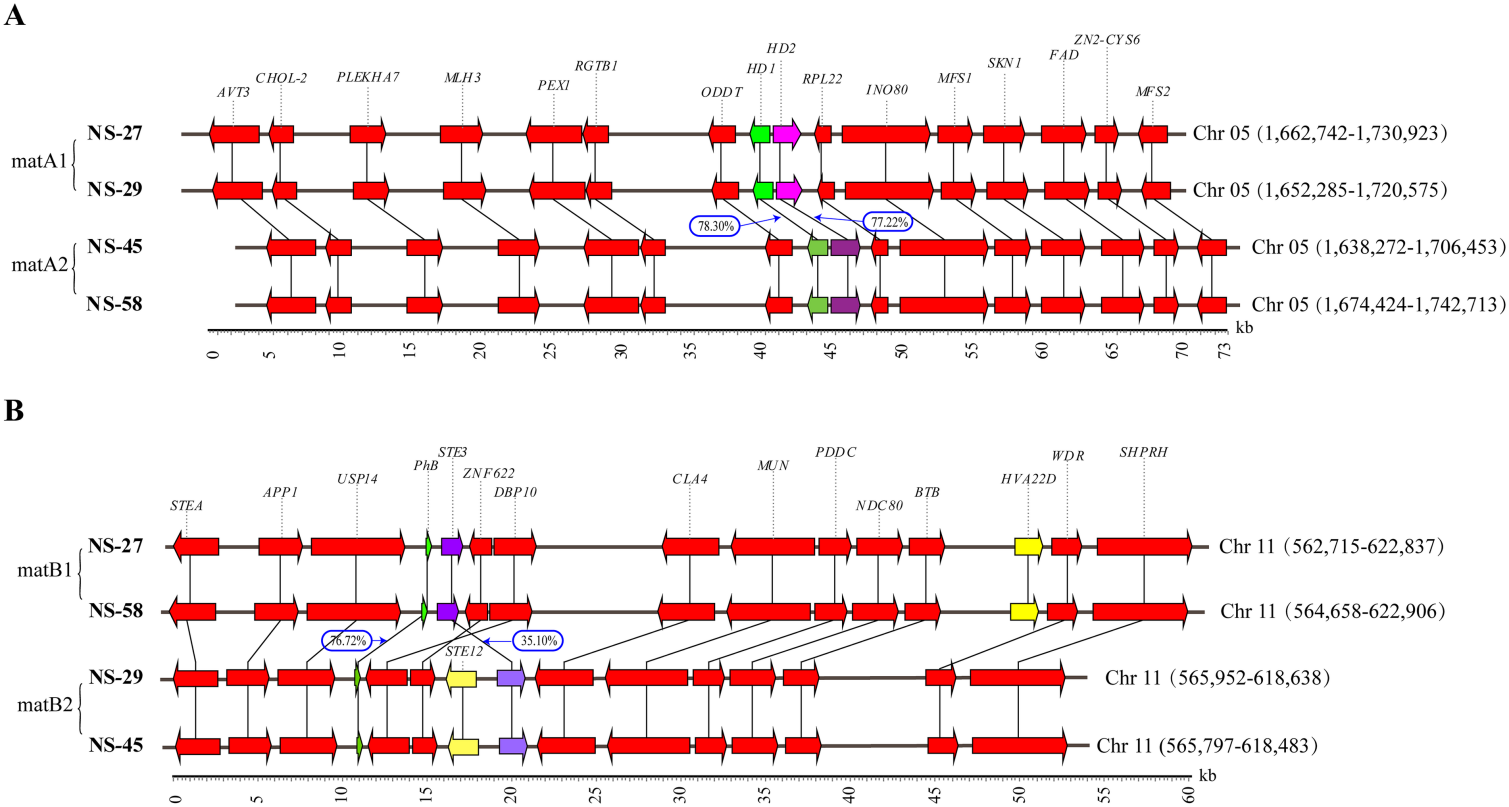
Distribution and collinearity of genes in the *matA* and *matB* loci of the four *N. sinensis* strains. **(A)** *MatA* loci of the four *N. sinensis* strains (*AVT3*, vacuolar amino acid transporter 3; *CHOL_2*, phospholipid methyltransferase; *PLEKHA 7*, pleckstrin homology domain-containing family A member 7; *MLH3*, DNA mismatch repair protein; *PEX1*, peroxisome biogenesis factor 1; *RGTB1*, geranylgeranyl transferase; *ODDT*, oxidoreductase; *HD1*, jomeobox KN domain; *HD2*, homeobox domain; *RPL22*, ribosomal L22e protein; *INO80*, ATP-dependent helicase; *MFS1*, MFS transporter; *SKN1*, beta-glucan synthesis-associated protein; *FAD*, ferric reductase NAD binding domain-domain-containing protein; *Zn2-Cys6*, fungal Zn^(2)^-Cys^(6)^ binuclear cluster domain; *MFS2*, major facilitator superfamily); **(B)** *MatB* loci of the four *N. sinensis* strains (*STEA*, STE like transcription factor, *APP1*, phosphatidate phosphatase; *USP14*, ubiquitin carboxyl-terminal hydrolase; *PhB*, pheromone precursor; *STE3*, pheromone receptor; *ZNF622*, cytoplasm protein; *DBP10*, ATP-dependent RNA helicase; *STE12*, transcription factor; *CLA4*, serine/threonine-protein kinase; *MUN*, homoaconitate hydratase; *PDDC*, pyridoxal-dependent decarboxylase; *NDC80*, probable kinetochore protein; *BTB*, sister chromatid cohesion protein; *HVA22D*, HVA22 family-domain-containing protein; *WDR*, WD repeat-containing protein; *SHPRH*, ubiquitin-protein ligase). The numbers in the blue box indicate the identity between corresponding genes.

This study focused on the comprehensive analysis of the A and B mating-type loci regions in four strains of *N. sinensis*. Specifically, the A mating-type locus region (approximately 73 kb) was located on chromosome 5 across all four strains, demonstrating high collinearity in the surrounding genes. This locus comprised the *HD1* and *HD2* genes, which were arranged in a head-to-head configuration. A comparative analysis of the HD1 and HD2 protein sequences among the four strains revealed that the sequences of strains NS-27 and NS-29 were identical and were designated as matA1, while the sequences of strains NS-45 and NS-58 were also identical and were designated as matA2. The sequence identity between matA1 and matA2 for the HD1 and HD2 proteins was 78.30 and 77.22%, respectively (Figure 5A; Supplementary Table S12).

The analysis of the B mating-type locus region (approximately 60 kb) revealed that the B mating-type loci in the four strains were also located on chromosome 11, exhibiting high synteny. Based on gene arrangement, the four strains can be categorized into two types: matB1 (NS-27 and NS-58) and matB2 (NS-29 and NS-45). The matB1 type comprised a closely adjacent pheromone precursor gene (*PhB*) and pheromone receptor gene (*STE3*). In contrast, the *PhB* and *STE3* gene regions in matB2 had undergone rearrangement and insertion events, with *ZNF622*, *DBP10*, and *STE12* inserted between these two genes; notably, *STE12* was absent at the matB1 locus. A comparison of the PhB and STE3 protein sequences between matB1 and matB2 indicated that the identity of PhB was 76.72%, whereas that of STE3 was only 35.10% (Figure 5B; Supplementary Table S12). Subsequently, Hi-C interaction heat maps of the B-mating-type region across four *N. sinensis* strains exhibited strong contact signals and continuous connectivity across gene-rearranged and inserted segments, with no detectable breakpoints (Supplementary Figure S5A). Meanwhile, alignment of HiFi reads to the corresponding genomes, visualized in Integrative Genomics Viewer (IGV), revealed average coverages of 43–70 × within rearranged/inserted regions for all four strains (Supplementary Figure S5B). These results corroborate the accuracy of the genome assemblies and demonstrate that the rearrangements and insertions in the B locus are genuine sequence features rather than assembly artefacts. In addition, we constructed separate phylogenetic trees for the STE3, STE12 and STEA proteins encoded within the B-mating-type region. The tree revealed that STE3 (matB1) and STE3 (matB2) form two distinct, well-supported clades (Supplementary Figure S6A). Comparative analysis showed that STE12 (PF02200.18, STE-like transcription factor) and STEA (PF00096.28, C2H2-type zinc finger) share similar functional domains, encoding 688 and 689 amino acids, respectively, with 43% domain coverage and 42.7% pairwise sequence identity. Phylogenetic analysis further indicated that the two proteins diverge into separate clades (Supplementary Figure S6B). In summary, the A mating-type locus of *N. sinensis* has maintained a conserved gene arrangement during long-term evolution, while the B mating-type locus exhibited greater diversity in both gene arrangement and sequence variation.

### Phylogenomic and evolutionary analysis

Orthologous-gene inventory across 23 Basidiomycota genomes highlights the unique genomic signature of *N sinensis*. All four monokaryotic strains (NS-27, NS-29, NS-45, NS-58) possess an almost identical catalogue of 1,615–1,617 single-copy orthologues—values that are statistically indistinguishable from those of closely related jelly fungi (e.g., *N. aurantialba*, *T. mesenterica*; 1,618–1,619) but are markedly higher than the out-groups *Ustilago hordei* (1,596) and *Wallemia mellicola* (1,609). Conversely, multi-copy and unclustered categories expand dramatically in the two out-group lineages (728–1,492 genes), reflecting the deeper phylogenetic distance and greater genome complexity of *Ustilaginomycotina* and *Wallemiomycotina* relative to the single-class *Tremellomycetes* data set. Thus, the orthologue profile corroborates the placement of *N. sinensis* within a tight *Tremellaceae* clade while providing a high-quality, low-redundancy gene set for downstream comparative and evolutionary analyses (Supplementary Figure S7 and Supplementary Table S14).

**Figure 6:**
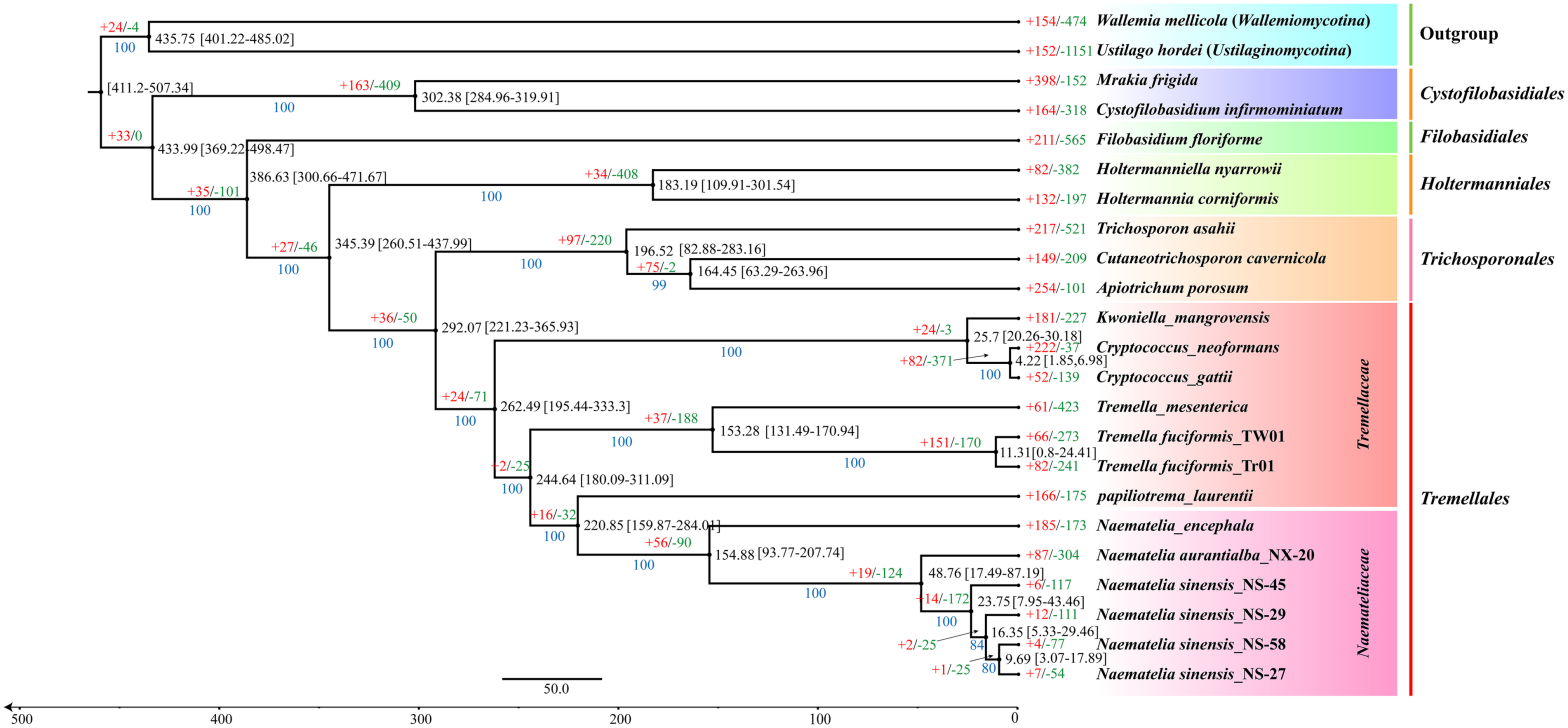
The evolutionary relationship and expanded and contracted gene families among 23 representative Basidiomycetes. The maximum likelihood method credibility tree was inferred from 1,407 single-copy orthologous genes. Blue numbers in the figure indicate bootstrap values from 1,000 replicates, while black numbers show node mean crown ages with 95% highest posterior density (HPD). Time unit is millions of years (Mya). The numbers of gene family expansion and contraction in each species are labeled after plus (in red color) and minus (in green color) symbols, respectively. The 23 fungal species comprise representatives from five different orders of the class *Tremellomycetes* (*Tremellales*, *Trichosporonales*, *Holtermanniales*, F*ilobasidiales,* and *Cystofilobasidiales*) and two outgroup taxa (*Wallemiomycotina* and *Ustilaginomycotina*).

To further investigate the genomic evolution within the genus *Naematelia*, a phylogenomic tree was constructed using 1,407 single-copy orthologous genes shared with 170,559 characters of amino acid residues delimited phylogenitc relationships among 23 fungal species with 80–100 bootstrap values (1,000 replicates). The results indicate that all five orders within the class *Tremellomycetes* are clearly delineated. Within the order *Tremellales*, species belonging to the families *Naemateliaceae* and *Tremellaceae* form two distinct clades. Our divergence time estimates indicate an ancient origin for the key lineages. Our divergence time analysis yielded the following estimates (Figure 6). The mean estimated crown age for the split between *Naemateliaceae* and *Tremellaceae* is approximately 220.85 Mya, with a broad 95% HPD of 159.78–284.01 Mya (spanning the late Carboniferous to early Jurassic). Within the genus *Naematelia*, species are estimated to have diversified at a mean crown age of 154.88 Mya (95% HPD: 93.77–207.74 Mya; Jurassic to early Cretaceous). Divergence among *N. sinensisstrains* is estimated to have occurred within a mean range of 9.96–23.75 Mya (95% HPD: 3.07–43.46 Mya; Miocene to Pliocene/Pleistocene). The considerable width of all confidence intervals underscores the substantial uncertainty in these estimates. Therefore, the precise timing should be considered preliminary, awaiting future studies with additional genomic data and fossil calibrations to reduce uncertainty and enable further validation.

In the evolutionary process of the 23 sampled fungal species, the gene family contraction occurred more common than the gene family expansion (Figure 6). Within the genus *Naematelia*, the species *N. encephala* and *N. aurantialba* exhibited pronounced gene family dynamics, with 185 and 87 expansions alongside 173 and 304 contractions, respectively. Among the four *N. sinensis* strains (NS-45, NS-29, NS-58, NS-27), gene family expansions were relatively fewer, totaling 6, 12, 4, and 7, while contractions numbered 117, 111, 77, and 54, respectively. Subsequent significance testing (*p* < 0.05) of these expansions and contractions in each *N. sinensis* strain identified 20 (1 expansion and 19 contractions), 18 (6 expansions and 12 contractions), 15 (3 expansions and 12 contractions), and 12 (4 expansions and 8 contractions) gene families exhibiting statistically significant shifts (Figure 6; Supplementary Table S15). Collectively, these data indicate that these gene families have undergone rapid and substantial evolutionary changes across the different *N. sinensis* strains. Gene screening from expanded and contracted gene families across the four strains revealed relatively modest numbers: 15–34 genes in expanded families and 9–32 genes in contracted families (Supplementary Table S15).

**Figure 7:**
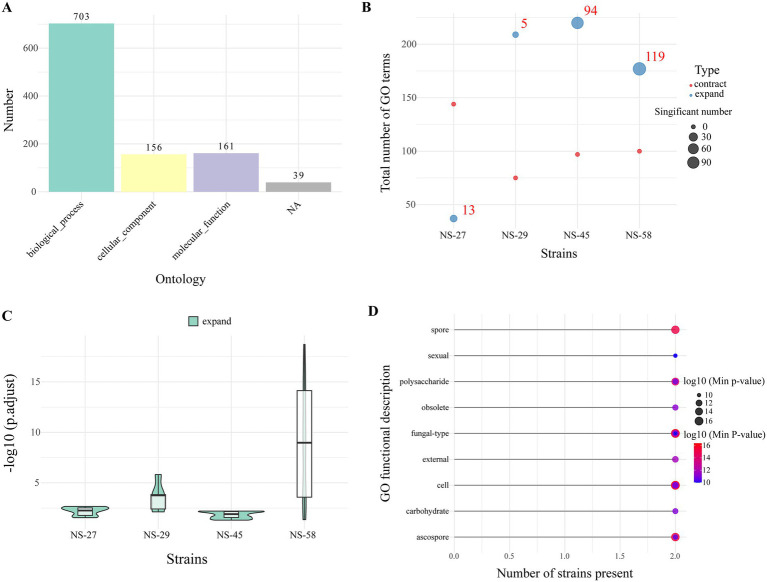
GO enrichment analysis of gene family expansion and contraction in the four strains. **(A)** Ontology distribution of GO terms. **(B)** GO term quantity distribution across the four strains. **(C)** *p*-value distribution of significant GO terms. **(D)** The top 20 most frequently shared GO functional enrichment analyses. Y-axis: GO functional descriptions (sorted in descending order of frequency); X-axis: number of strains exhibiting each function; The size and color of dots represent –log_10_ (minimum *p*-value), indicating the statistical significance of enrichment. Larger dots/deeper blue color denotes more significant enrichment.

Integrated GO functional enrichment analysis of expanded and contracted gene families from the four strains demonstrated that Biological Process constituted the predominant enrichment category, encompassing 703 significant GO terms and representing the vast majority of total enrichment results. In contrast, Cellular Component and Molecular Function categories showed relatively limited enrichment (Figure 7A). Comparative analysis of GO enrichment patterns among strains revealed distinct differential profiles: contracted gene families showed no significant GO term enrichment across all strains, whereas expanded gene families exhibited strain-specific patterns, with NS-58 and NS-45 displaying 119 and 94 significantly enriched GO terms, respectively (Figure 7B). The distribution of adjusted *p*-values further illustrated functional enrichment significance differences: NS-58 exhibited the broadest significance range (approximately 2–16), encompassing multi-level functions from marginally significant to highly significant; NS-27 and NS-45 showed significance concentrated in the moderate-to-high range (approximately 4–12); while NS-29 displayed a relatively concentrated significance distribution (Figure 7C). Analysis of the top 20 most frequently shared functional terms revealed that spore development-related functions, particularly ascospore formation, represented the most prevalent shared GO terms, showing significant enrichment across multiple strains with exceptionally high statistical significance (−log₁₀(p) ≈ 16). Additionally, carbohydrate metabolism, cell wall synthesis, and sexual reproduction-related functions appeared with high frequency across multiple strains (Figure 7D). These shared functions constitute the core functional modules of expanded genes in *N. sinensis* strains, revealing conserved characteristics during population evolutionary processes.

## Discussion

### Taxonomic study for Jin Er

The taxonomic boundaries of Jin Er have remained ambiguous and have long been a subject of debate. It was previously classified as *Tremella mesenterica* (Luo et al., 1987) and *T. aurantialba* (Bandoni and Zang, 1990). A previously published phylogenetic tree reconstruction based on seven genetic markers clarified the evolutionary relationships within *Tremellomycetes*, which led to the renaming of the species as *Naematelia aurantialba* (Liu et al., 2015). By 2024, Tang and Yang (2024) identified four species (including three newly described ones) from southwestern China, based on morphological traits, molecular phylogenetic data, and geographic distribution patterns. These species were designated as *N. sinensis*, *N. aurantialba*, *N. nodulosa*, and *N. pedicellata*, respectively. Among them, the commonly cultivated species was *N. sinensis.* In this study, we constructed a phylogenetic tree using the maximum likelihood method based on ITS sequences. The resu6lts demonstrated that our four strains, along with the *N. aurantialba* NX-20 strain (Sun et al., 2021), clustered within the *N. sinensis* clade (Supplementary Figure S9), which was consistent with the findings reported by Tang and Yang (2024). Consequently, *N. sinensis* was adopted as the scientific name for “Jin Er” in this study.

### Genome sequencing, assembly, and genomic component prediction

In this study, a combination of Illumina and HiFi sequencing technologies was utilized, complemented by Hi-C-assisted assembly, to construct four haploid genomes derived from the monokaryons of a commercial *N. sinensis* cultivar. The resulting monokaryotic genomes demonstrated significantly higher levels of completeness compared to those previously reported for other strains (Table 2). Through the application of Hi-C technology, we successfully assembled the genomes of four distinct mating types of *N. sinensis* monokaryotic strains into 12 chromosomes each, with varying lengths (Figure 2A and Supplementary Table S5). Each chromosome terminated with two telomeres, yielding a total of 24 telomeres per strain (Figure 2B and Supplementary Table S6), which was consistent with the 12 chromosomes observed under confocal microscopy (Figure 2C). These findings confirmed the successful assembly of four chromosomal-level *N. sinensis* genomes, each consisting of 12 chromosomes. Additionally, we benchmarked two state-of-the-art HiFi-only assemblers, NextDenovo and Hifiasm, using the same set of PacBio HiFi reads. Surprisingly, NextDenovo consistently yielded more contiguous and complete assemblies than Hifiasm across all four monokaryons (Supplementary Table S2). This finding contradicts the prevailing expectation that Hifiasm would outperform in the context of high-fidelity long reads. We attribute this unexpected advantage to the low heterozygosity and relatively straightforward repeat landscape of the *N. sinensis* genome, which enables NextDenovo’s ‘correct-then-assemble’ strategy to leverage the high accuracy of HiFi reads more effectively, resulting in cleaner unitigs with fewer false duplications. Our results indicate that for organisms exhibiting similarly low genomic complexity, NextDenovo can serve as a complementary or even preferable alternative to Hifiasm.

The analysis of repetitive sequences in the genomes of four strains revealed that these sequences constituted approximately 4.2 to 4.6% of the total genomic content. This primarily included simple repeats, which account for about 2.5%, and long terminal repeats (LTRs), ranging from 1.02 to 1.56%. Notably, the Gypsy gene family predominated among the LTRs, representing 0.9 to 1.30% of the genomes (Supplementary Table S7). These findings were consistent with those reported for the NX-20 strain in previous studies (Sun et al., 2021). Comparative analysis indicated that the repetitive sequences in the genome of *N. sinensis* were significantly fewer than those observed in other fungal species, such as *T. fuciformis* (approximately 10.7%) (Li et al., 2023), *L. edodes* (approximately 31%) (Yu et al., 2022), and *Wolfiporia hoelen* (approximately 48%) (Li S. J. et al., 2022; Li S. W. et al., 2022). Furthermore, 41–53 non-coding RNAs were predicted in the four *N. sinensis* genomes, showing a marked discrepancy compared to the previously reported result of 67 RNAs (Sun et al., 2021). The differences were predominantly concentrated in ribosomal RNA (rRNA) and transfer RNA (tRNA). Specifically, the NX-20 strain was predicted to possess 44 tRNAs, whereas our four strains were predicted to contain only 25 tRNAs. Notably, the NS-58 strain exhibited 6–13 more rRNAs compared to the other strains (Supplementary Table S8). These variations were likely attributable to potential differences in chromosomal structures among the various strains, including deletions, duplications, inversions, or translocations. Such structural variations might lead to the loss, gain, or positional alterations of non-coding RNA genes, thereby influencing the abundance of non-coding RNAs. Additionally, no microRNAs (miRNAs) were predicted in any of the five strains due to the absence of a basidiomycetes miRNA database (Qu et al., 2016).

### Comparative genomics analysis

In this study, a collinearity analysis to investigate structural variations among five strains of *N. sinensis* was utilized based on coding genes (Figure 3). The results demonstrated that the four *N. sinensis* strains exhibited highly syntenic regions relative to strain NX-20. This finding aligned with the collinearity analysis of *L. edodes* strain genomes reported in prior studies (Yu et al., 2022). Furthermore, a high degree of collinearity was observed between the two *T. fuciformis* strains. Collinearity comparisons between *T. fuciformis* and *N. sinensis* revealed several inversions and rearrangements within homologous regions (Figure 3). These findings suggested that chromosomes might have undergone a series of fusions or breakages during the long evolutionary history of *N. sinensis* and *T. fuciformis*. Additionally, pairwise synteny analyses were conducted for the genomes of four *N. sinensis* strains based on nucleic acid sequences (Supplementary Figures S3, S4).

Comparative synteny analyses among strains revealed that the genomes of the four *N. sinensis* strains, which exhibited distinct mating types, were highly conserved, with no substantial structural variations detected among them. It is important to note that this conclusion is based on the comparison of a limited number of strains derived from a commercial cultivar; thus, the observed structural conservation applies specifically to this lineage, and broader inferences regarding species-wide genome stability would require analysis of a more diverse set of isolates. We conducted an in-depth analysis of the unique genes present in the genomes of the four *N. sinensis* strains and identified 6,019 common genes shared across these strains. Notably, a unique gene was found exclusively in the N-58 strain (Figure 4C). Furthermore, comparisons between sexually compatible strains demonstrated that the NS-27 and NS-45 strains shared 6,179 common genes, with 180 and 148 unique genes, respectively (Figure 4D). Similarly, the NS-29 and NS-58 strains shared 6,169 common genes, accompanied by 169 and 163 unique genes, respectively (Figure 4E). These findings paralleled the results of unique gene analyses reported for the SP3 and SP30 strains of *L. edodes* in previous studies (Yu et al., 2022).

In this study, comparative genomic analysis of CAZyme families across four *N. sinensis* strains identified 225–229 CAZymes per genome (Figure 4E), a range consistent with that previously reported for strain NX-20 (207 CAZymes) (Sun et al., 2021). The CAZyme complement in *N. sinensis* was notably smaller than that of its fungal host, *Stereum hirsutum*, which encodes 560 CAZymes. Such a reduction in CAZyme repertoire is commonly reported in *Tremellales* species (Akapo et al., 2019) and may reflect an evolutionary trend toward reduced carbohydrate-degrading capacity in fungi that rely nutritionally on a host. This pattern is often described in the literature as convergent evolution, yet the underlying host-fungus interaction mechanisms remain largely unclear. Therefore, rather than interpreting it as a specific adaptation to preserve host cell walls, it more likely reflects a trend of genomic streamlining resulting from nutritional dependence. We emphasize that these findings represent a comparative genomic correlation; direct experimental evidence linking the observed CAZyme reduction to specific host interaction mechanisms is still lacking.

Similarly, the cytochrome P450 (CYP450) superfamily, comprising ferrous heme thiolate proteins involved in detoxification and secondary metabolism (Bhattacharyya et al., 2014), showed reduced copy numbers in *N. sinensis* (10–11 genes) compared to wood-decaying fungi such as *S. hirsutum* (536 genes). This reduction parallels patterns observed in other *Tremellales* species, including *T. mesenterica* (8 genes) and *N. encephala* (10 genes) (Akapo et al., 2019), as well as in *Saccharomycotina* yeasts inhabiting nutrient-rich environments (Kgosiemang et al., 2014). These convergent patterns across phylogenetically distant lineages suggest that streamlined metabolic repertoires may reflect evolutionary adaptation to host-derived simple carbon sources (Aliyu et al., 2020). However, we caution that such genomic correlations do not demonstrate functional mechanisms of host interaction or parasitism; rather, they highlight evolutionary trends associated with nutritional specialization. Variations in P450 counts between our strains and the NX-20 strain (26 genes) (Sun et al., 2021) may additionally reflect technical differences in genome assembly and annotation methodologies.

### A and B mating type locus of *N. sinensis*

In Basidiomycota, mating-type genes constitute the most direct indicators of how two nuclei coordinate to complete the reproductive life cycle. For species that require mating between monokaryons carrying complementary mating-type alleles, sexual reproduction is achieved through reciprocal exchange of diffusible pheromones (P/R locus) and post-fusion interactions between homeodomain proteins (HD) (Coelho et al., 2017). We previously confirmed the tetrapolar mating system of *N. sinensis* by laboratory mating tests (Cao et al., 2024) and analysed the A and B mating-type loci using genome-resequencing data (Shen et al., 2024).

Focusing on four monokaryotic strains, we now provide a detailed structural comparison of both loci. At the A mating-type locus, a single *HD1/HD2* gene pair resides on Chr05, flanked upstream and downstream by the conserved *ODDT* and *PRL22* genes (Figure 5A). Notably, the gene order of the *N. sinensis* A locus is highly conserved relative to closely related species such as *N. encephala* and *T. mesenterica* (Shen et al., 2024), indicating ancestral synteny within *Tremellaceae*. In contrast, *mip* and *β-fg* are neither adjacent to *HD1/HD2* nor genetically linked, differing from the tight *mip–HD–β-fg* arrangement typical of most edible *Agaricomycetes* (Bao, 2019). Comparative analysis of the encoded proteins revealed that NS-27 and NS-29 share identical HD1/HD2 sequences (designated matA1), whereas NS-45 and NS-58 carry an alternative allelic form (matA2). Pairwise identities between matA1 and matA2 are 78.30% (HD1) and 77.22% (HD2) (Figure 5A), consistent with allelic divergence maintained by balancing selection.

The contrasting evolutionary patterns between A and B mating-type loci in *N. sinensis* reflect fundamental differences in selective pressures. While the A locus maintains high conservation, likely due to its role in essential developmental pathways, the B locus exhibits remarkable structural diversity. This pattern aligns with observations in other basidiomycetes where B loci evolve as “evolutionary hotspots” driving mating specificity diversification. The insertion of *STE12* between pheromone genes in matB2 types, absent in matB1, exemplifies how these regions can accommodate novel genetic elements that may contribute to enhanced recognition specificity between compatible mating partners. The identified gene inversions and rearrangements within the B mating-type locus, particularly the reorientation of DBP10 and ZNF622 homologs between matB1 and matB2 types, likely arose from DNA double-strand break repair mechanisms rather than transposable element activity. The absence of repetitive elements near breakpoint regions supports this hypothesis (Yang et al., 2025). Such rearrangements may contribute to recombination suppression around mating-type loci, promoting allelic diversification - a mechanism thought to drive the evolution of mating specificity (Lucotte et al., 2025). This structural plasticity contrasts sharply with the conserved A locus, suggesting different evolutionary trajectories for these two essential components of the fungal mating system. Despite the structural rearrangements, the B locus gene order in *N. sinensis* shows strong conservation with phylogenetically related species such as *N. encephala* and *T. mesenterica* (Shen et al., 2024; Yang et al., 2025). This conservation suggests that the core functional components of the B locus are maintained across species boundaries, while structural variations may contribute to species-specific mating recognition. The low sequence identity of STE3 proteins (35.10%) between matB1 and matB2 types, compared to higher conservation of PhB (76.72%), indicates differential evolutionary rates among B locus components, with receptor genes evolving more rapidly to maintain mating specificity through diversifying selection.

### Phylogenomic and evolutionary analysis

The four *N. sinensis* monokaryotic strains (NS-27, NS-29, NS-45, NS-58) shared a highly conserved set of 1,615–1,617 single-copy orthologs, comparable to closely related jelly fungi (*N. aurantialba* and *T. mesenterica*) and substantially higher than outgroup species (*U. hordei* and *W. mellicola*). This genomic conservation reflects the close phylogenetic relationship and common origin of these strains, all derived from the same commercial cultivar. The stable genomic architecture may be associated with a host-dependent lifestyle, where constant nutrient supply from the obligate host (*S. hirsutum*) potentially reduces selection pressure for gene innovation and narrow ecological niches constrain genomic expansion. However, these interpretations remain tentative given the limited strain sampling. While the observed stability supports the phylogenetic placement of *N. sinensis* within *Naemateliaceae*, broader inferences regarding species-level evolutionary stability or the fixation of parasitic adaptations require analysis of more diverse isolates.

Comparative genomics revealed that gene-family contraction substantially outnumbered expansion in *N. sinensis* (Figure 6). he four strains exhibited only 4 to 12 expanded gene families, whereas the number of contracted gene families ranged from 54 to 117 (Supplementary Table S15). This contraction-biased pattern aligns with niche-specialization theory and is consistent with genome evolution trends reported across basidiomycetes, where gene loss events are more prevalent than gene gain events in yeast-like lineages (Nagy et al., 2014; Nagy, 2017). Similar evolutionary streamlining has also been documented in the subphylum *Saccharomycotina*, where fungi thriving in simple nutrient environments exhibit pronounced genome reduction (Kgosiemang et al., 2014). In *N. sinensis*, parasitic specialization likely drives the loss of redundant genes to optimize energy allocation, while the stable rotten-wood habitat reduces selection for adaptive gene expansion. Over time, such specialization can lead to substantial genetic material loss as fungi adapt to host-derived simple carbon sources, often resulting in reduced genome size (Aliyu et al., 2020). Thus, the contraction-biased trajectory in *N. sinensis* reflects a broader evolutionary tendency toward genome streamlining and functional optimization rather than innovation—a strategy that may underpin its competitive adaptation to a narrow, nutrient-stable ecological niche.

Analysis of the most frequently shared GO terms identified spore-development functions—especially ascospore formation—as the most significantly enriched shared category (Figure 7D), appearing frequently across multiple strains. Carbohydrate metabolism, cell-wall synthesis, and sexual-reproduction functions were also highly conserved. These results demonstrate that, despite its narrow niche, *N. sinensis* has retained strong conservation of reproduction-related functions, likely ensuring population persistence in a stable habitat. Enhanced spore development may facilitate effective dispersal and establishment in the cool and moist environment, while conserved reproductive modules support population stability under obligate parasitism. Future studies integrating host-interaction omics will help elucidate the evolutionary mechanisms underlying this pattern of conservation coupled with micro-differentiation and its ecological significance. Future research could elucidate the evolutionary mechanisms and ecological adaptive significance underlying the coexistence of conserved and divergent patterns in *N. sinensis* by integrating multi-omics studies of host interaction, encompassing genomics, transcriptomics, metabolomics, and proteomics.

## Conclusion

This study successfully generated high-quality chromosome-level genome assemblies of *N sinensis* and systematically elucidated its evolutionary history and genomic features, thereby validating the two primary hypotheses proposed. These outcomes significantly enrich the foundational knowledge of this important fungal species. Firstly, by integrating PacBio HiFi long-read sequencing with Hi-C scaffolding, we achieved highly contiguous assemblies of four monokaryotic strains representing different mating types, each comprising 12 chromosomes with an approximate genome size of 21 Mb. This represents a marked improvement over previously reported genomes within *Tremellaceae* and establishes a solid foundation for investigating complex mating-type loci and key gene families. Secondly, comparative genomics and phylogenomic analyses precisely positioned *N. sinensis* within the *Naemateliaceae* family and revealed distinctive genomic features, including chromosomal rearrangements and contractions in certain gene families. These genomic patterns are consistent with a potential association with its parasitic lifestyle and provide a foundation for exploring its underlying adaptive mechanisms, though causal relationships remain to be functionally validated. The observed diversity in mating-type locus architecture further advances understanding of its reproductive strategies. In summary, this work not only delivers the first comprehensive, high-quality genomic resource for *N. sinensis* but also lays essential groundwork for future studies on its genetics, evolutionary biology, and breeding improvements, thereby facilitating its application in biotechnology and edible mushroom cultivation.

## Data Availability

The datasets presented in this study can be found in online repositories. The names of the repository/repositories and accession number(s) can be found in the article/Supplementary material.

## Footnotes

1
https://github.com/Nextomics/NextDenovo

2
http://www.ebi.ac.uk/about/vertebrate-genomics/software/

3
http://p450.riceblast.snu.ac.kr/index.php?a=view

4
https://www2.unil.ch/biomapper/chromomapper/

5
www.omicshare.com/tools
