# Supplementary figures and images for: Chromosome-scale genome assembly of Naematelia sinensis (Jin Er mushroom) offers new insights into the distinct mating-type genomes feature and genome evolution
Source: Front Microbiol. 2026 Apr 2;17:1669303. doi: 10.3389/fmicb.2026.1669303 (PMC13085929; doi:10.3389/fmicb.2026.1669303)

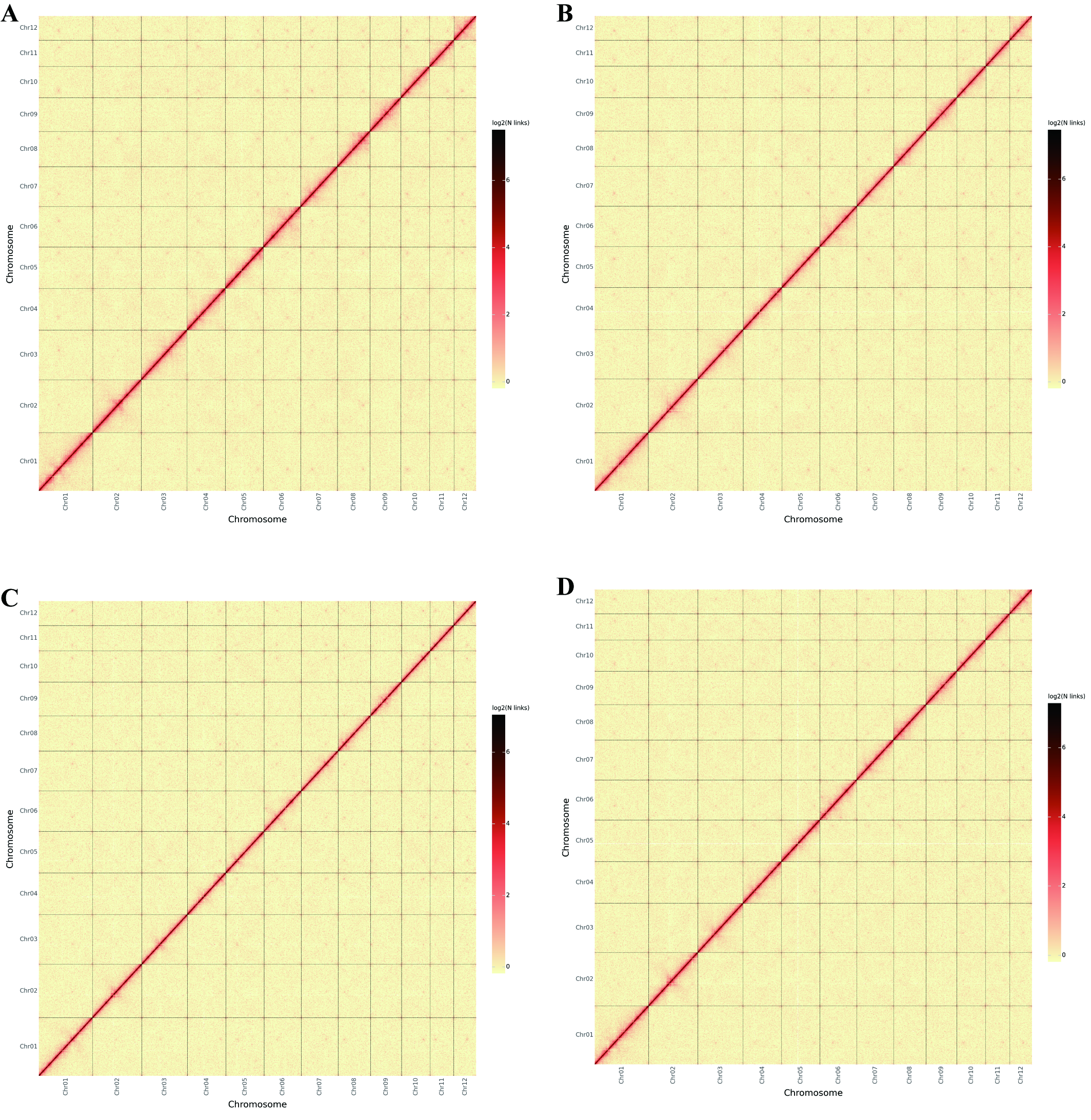

Supplement: Supplementary file 2 [file Image_2.tif]

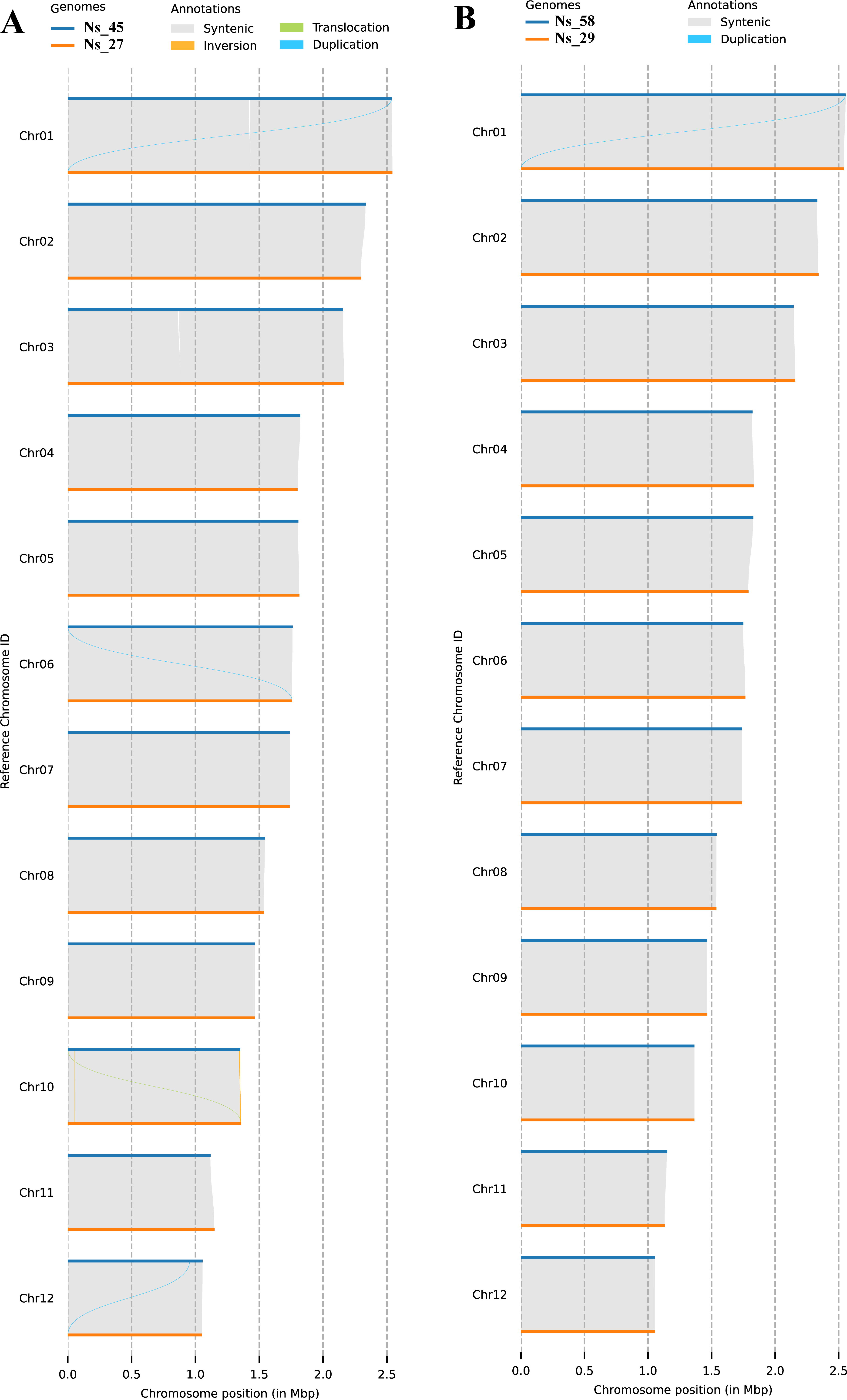

Supplement: Supplementary file 3 [file Image_3.tif]

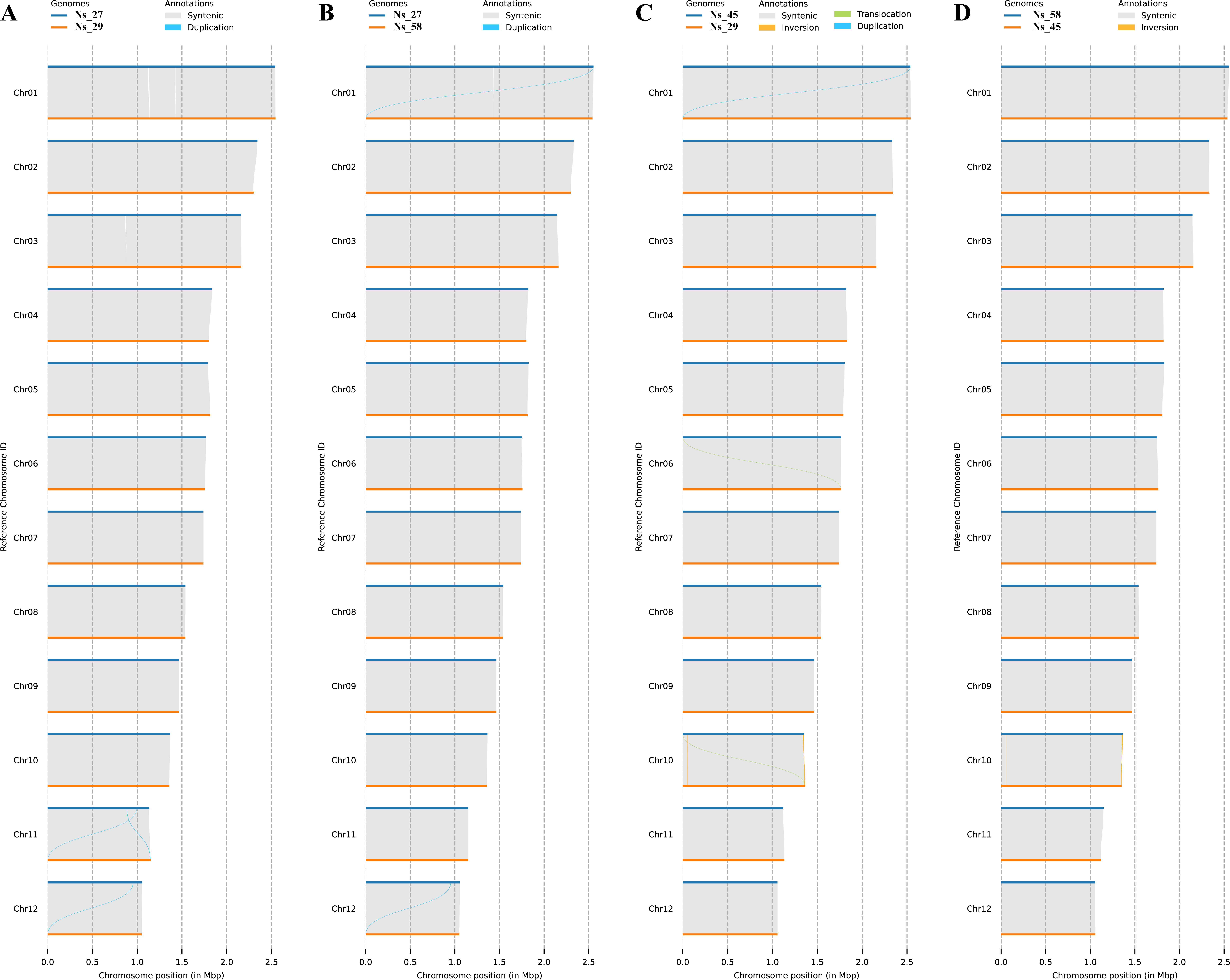

Supplement: Supplementary file 4 [file Image_4.tif]

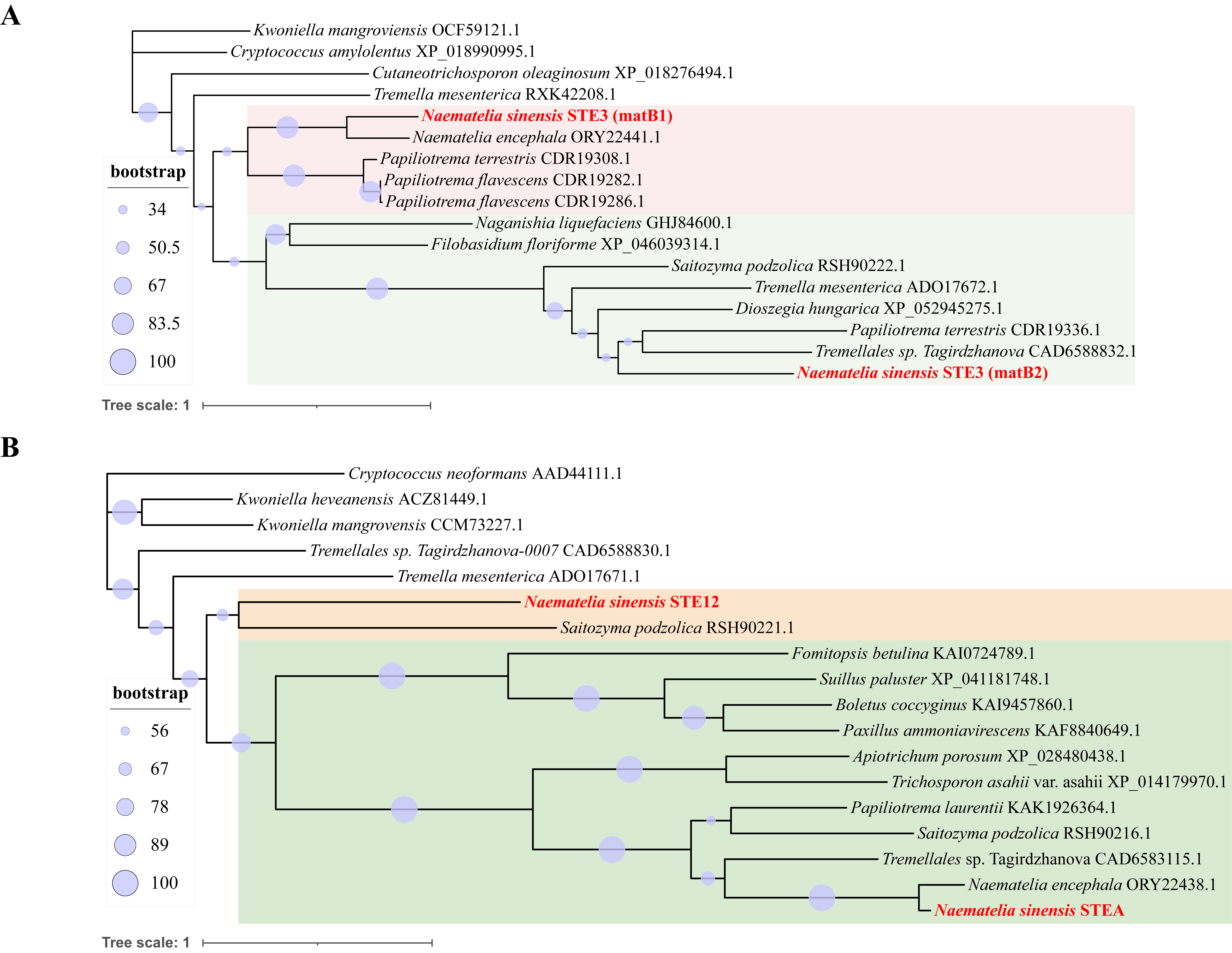

Supplement: Supplementary file 6 [file Image_6.tif]

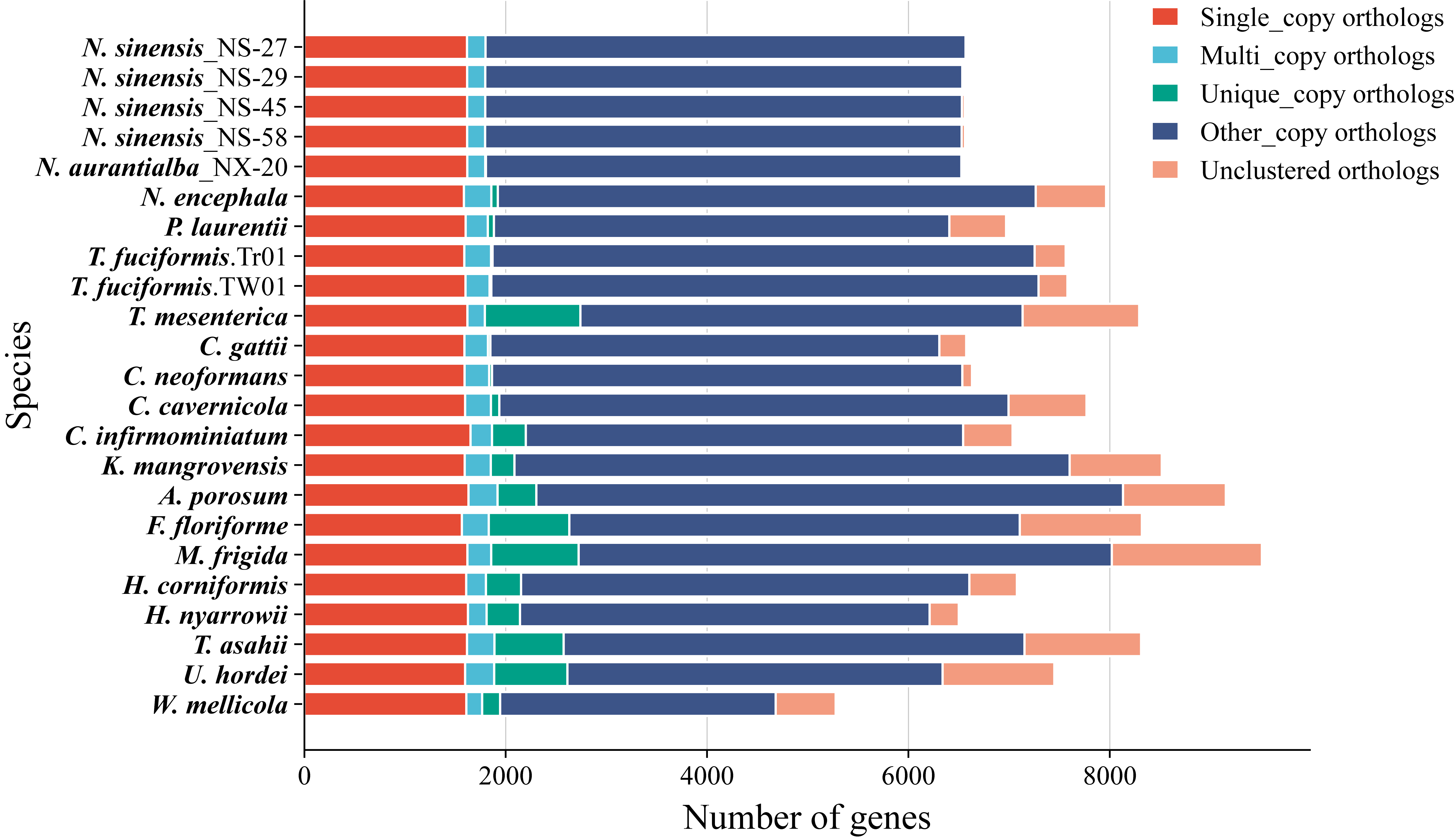

Supplement: Supplementary file 7 [file Image_7.tif]

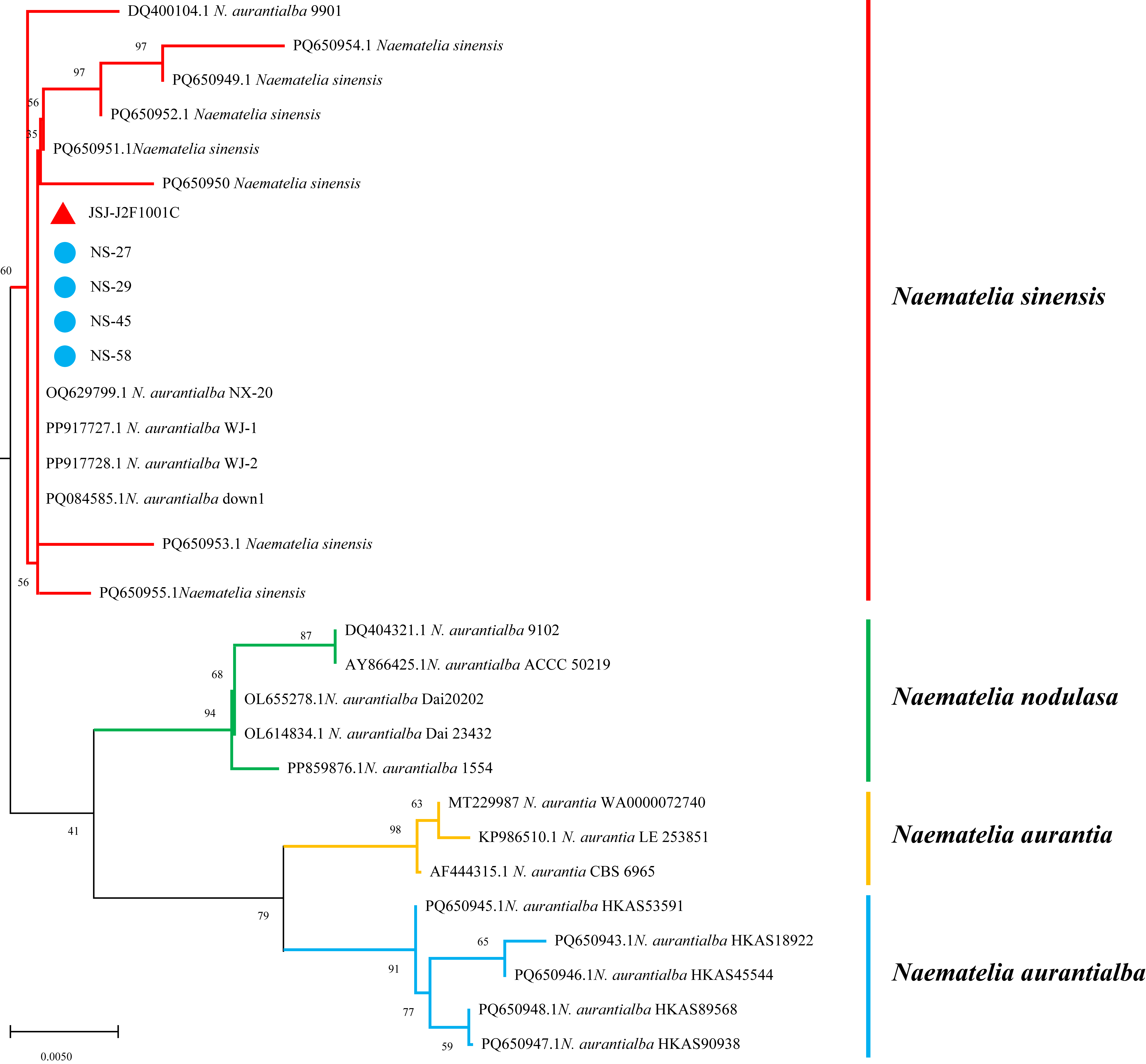

Supplement: Supplementary file 8 [file Image_8.tif]
